# A Fast DFA Algorithm for Multifractal Multiscale Analysis of Physiological Time Series

**DOI:** 10.3389/fphys.2019.00115

**Published:** 2019-03-01

**Authors:** Paolo Castiglioni, Andrea Faini

**Affiliations:** ^1^IRCCS Fondazione Don Carlo Gnocchi, Milan, Italy; ^2^Department of Cardiovascular Neural and Metabolic Sciences, Istituto Auxologico Italiano, IRCCS, S.Luca Hospital, Milan, Italy

**Keywords:** hurst exponent, multiscale analysis, multifractality, HRV, EEG

## Abstract

Detrended fluctuation analysis (DFA) is a popular tool in physiological and medical studies for estimating the self-similarity coefficient, α, of time series. Recent researches extended its use for evaluating multifractality (where α is a function of the multifractal parameter *q*) at different scales *n*. In this way, the multifractal-multiscale DFA provides a bidimensional surface α(*q,n*) to quantify the level of multifractality at each scale separately. We recently showed that scale resolution and estimation variability of α(*q,n*) can be improved at each scale *n* by splitting the series into maximally overlapped blocks. This, however, increases the computational load making DFA estimations unfeasible in most applications. Our aim is to provide a DFA algorithm sufficiently fast to evaluate the multifractal DFA with maximally overlapped blocks even on long time series, as usually recorded in physiological or clinical settings, therefore improving the quality of the α(*q,n*) estimate. For this aim, we revise the analytic formulas for multifractal DFA with first- and second-order detrending polynomials (i.e., DFA_1_ and DFA_2_) and propose a faster algorithm than the currently available codes. Applying it on synthesized fractal/multifractal series we demonstrate its numerical stability and a computational time about 1% that required by traditional codes. Analyzing long physiological signals (heart-rate tachograms from a 24-h Holter recording and electroencephalographic traces from a sleep study), we illustrate its capability to provide high-resolution α(*q,n*) surfaces that better describe the multifractal/multiscale properties of time series in physiology. The proposed fast algorithm might, therefore, make it easier deriving richer information on the complex dynamics of clinical signals, possibly improving risk stratification or the assessment of medical interventions and rehabilitation protocols.

## Introduction

In the mid of the 90s, the algorithm of detrended fluctuation analysis (DFA) gave a great boost to the study of fractal physiology providing an easy-to-calculate method for evaluating the Hurst's exponent of physiological times series (Castiglioni et al., 2017). DFA estimates the slope α of the log-log plot of a fluctuations function, *F(n)*, over a range of time scales *n*. The fluctuations function is the root mean square, or second-order moment, of the deviations of blocks of *n* data from a polynomial trend. Due to the intrinsic variability of the *F(n)* estimate, α is calculated as the slope of the least-square regression line fitting log *F(n)* on log *n*.

DFA became quickly popular in the field of heart rate variability analysis and since its first applications the *F(n)* of heart rate was described by two slopes, one at the shorter (*n* < 16) and one at the longer scales (Peng et al., 1995). Then, similar multi-slope approaches were proposed for electroencephalogram (EEG) recordings (Hwa and Ferree, 2002; Jospin et al., 2007). The idea that α may change with the scale *n* and that the resulting α(*n*) profile may provide information on physiological or pathophysiological mechanisms was further investigated by different groups in the following years. Continuous α(*n*) profiles from noisy *F(n)* estimates were obtained by applying a simplified Kalman filter whose coefficients, however, were defined empirically, making the procedure somehow arbitrary (Echeverria et al., 2003). Other authors extended the two-slope approach fitting the regression line over a short running window (Gieraltowski et al., 2012; Xia et al., 2013): in this way a continuous α(*n*) profile was obtained as the central point of the window, *n*, moved from the shortest to the longest scale. The running-window approach was also applied to study multifractality. Multifractal series are composed by interwoven fractal processes and the multifractal DFA approach extends the calculation of the second-order fluctuations function to a range of positive and negative moments that includes *q* = 2 (the second-order moment): in fact, positive *q* moments amplify the contribution of fractal components with larger amplitude and negative *q* moments the contribution of fractal components with smaller amplitude (Kantelhardt et al., 2002). This led to the calculation of the local slope of the *q*^th^-order fluctuation function, α(*q,n*), as the slope of the regression lines fitting *F*_*q*_*(n)* over a running window at each *q* separately (Gieraltowski et al., 2012). It should be considered that the scale resolution achievable with the regression line method is limited because the window width cannot be too short to make α insensitive to the estimation variability.

A third approach is based on estimating the slope as the first derivative of log *F(n)* (Castiglioni et al., 2009, 2011a). Assuring higher α(*n*) resolution, it allows obtaining a more detailed description of the deviations of log *F(n)* from the straight line, which resulted useful for describing the autonomic integrative control (Castiglioni et al., 2011a) and its alterations (Castiglioni and Merati, 2017). However, this approach requires minimizing the variability of the *F(n)* estimator because of its sensitivity to noise. Traditional DFA estimators calculate *F(n)* splitting the series into consecutive non-overlapped blocks. If the series is split into maximally overlapped blocks, which means that consecutive blocks of size *n* have *n*-1 samples in common, the variability of the *F(n)* estimator decreases substantially, allowing the evaluation of the slope as the first derivative (Castiglioni et al., 2018). A drawback of this approach is the much higher computational load that may make unfeasible the analysis of long series of data, as often occur in heart-rate variability or EEG studies. The computational load is even greater for calculating *F*_*q*_*(n)* and the multifractal-multiscale spectrum, α(*q,n*).

Strictly related to the determination of the local slope of *F*_*q*_*(n)* is the identification of number and position of crossover scales where the fractal properties change. Crossovers may be due to interferences affecting the measures (Ludescher et al., 2011) or may reflect specific aspects of the physiological system generating the series, like short-range correlations (Höll and Kantz, 2015). Statistical models for identifying crossover points in *F*_*q*_*(n)* are based on fitting piecewise regressions and on iterative hypothesis testing (Ge and Leung, 2013). Other approaches identify the scales where the *F*_*q*_*(n)* curves change their slope by introducing the focus-based multifractal formalism (Mukli et al., 2015): accordingly, if one assumes a bimodal scale dynamics, the multifractal fluctuations function is directly modeled by one or two fan-like structures in which log-log straight lines, each corresponding to an order *q*, may converge to a focus at the largest scale. Multifractal crossovers are then decomposed by an iterative process that minimizes the residual errors of least-square fittings (Nagy et al., 2017; Mukli et al., 2018). Clearly, the estimation variability of the *F*_*q*_*(n)* curves negatively affects the identification of the focuses and decreases the overall statistical power of these methods. Therefore, also these methods can take advantage of the use of maximally overlapped blocks for improving the *F*_*q*_*(n)* estimates and for better modeling the multifractal dynamics.

**Figure 1:**
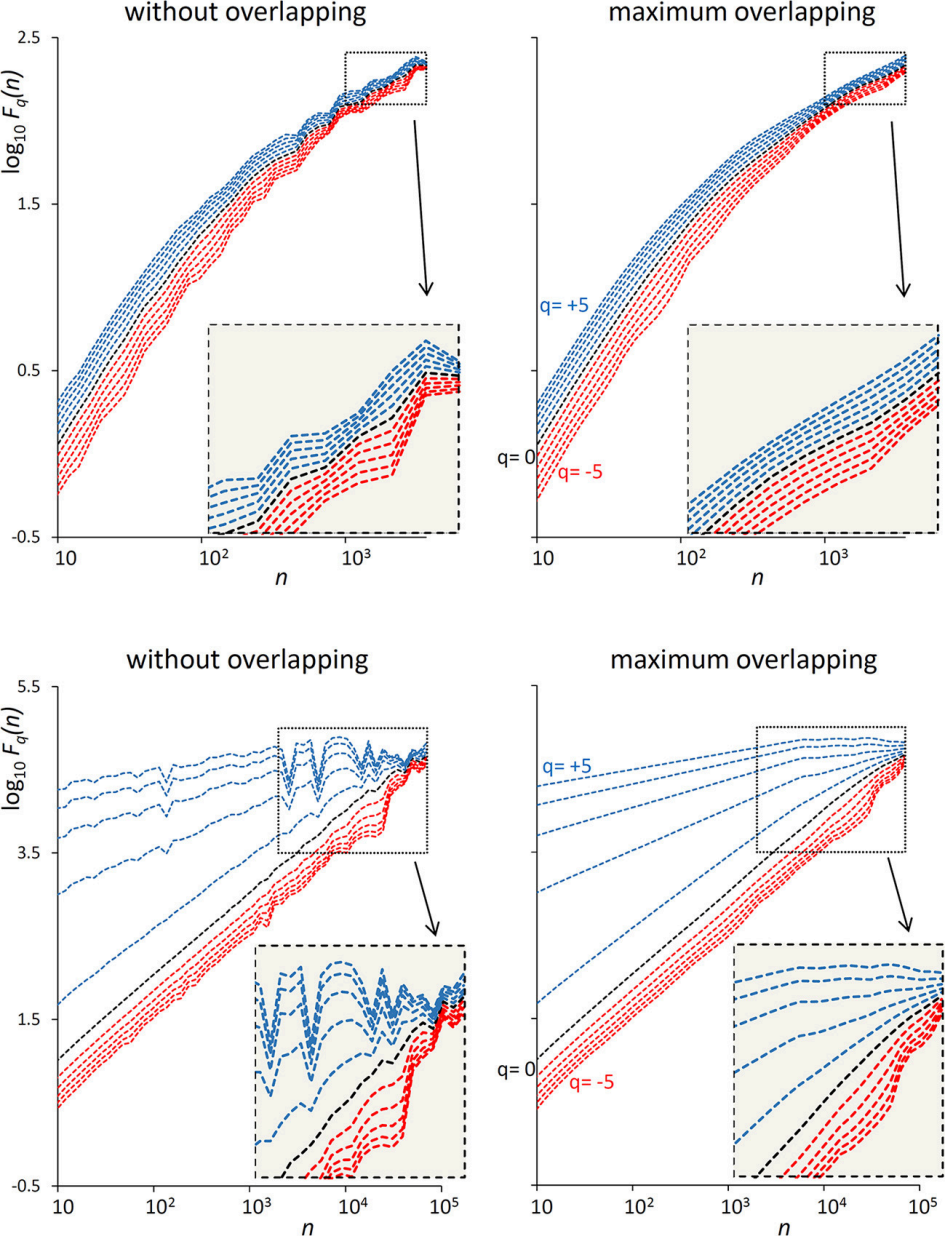
Effect of blocks overlapping on variability and computation time of *F*_*q*_*(n)* estimates. **(Upper)** DFA_1_ fluctuation functions for an autoregressive series {*x*_*i*_} of *N* = 16,384 samples generated as *x*_*i*_ = *ax*_*i*−1_+*wn*_*i*_ where *wn*_*i*_ is white Gaussian noise with zero mean and unit variance and *a* = 0.9391014 as in Kiyono (2015), corresponding to a low-pass filter with cut-off frequency *f*_*co*_ = 0.01, or a cross-over scale *n*_*co*_ = 100 (Höll and Kantz, 2015); calculation time for estimating *F*_*q*_*(n)* over 38 block-sizes *n* was 4 s without overlapping and 3 min with maximum overlapping on a personal computer. **(Lower)** DFA_1_ fluctuation functions for *N* = 300,000 random samples with Cauchy distribution (location 0, scale 3); calculation time for estimating *F*_*q*_*(n)* over 55 block-sizes *n* was 58 s without overlapping, 5 h and 22 min with maximum overlapping.

The examples of Figure 1 illustrate the reduction of estimation variability and the related computational costs when using maximum overlapping. In the example of a first-order autoregressive process generated by white Gaussian noise (Figure 1, Upper), the variability of the *F*_*q*_*(n)* estimate without overlapping might suggest the presence of a focus at the larger scales. A focus should not be present, because at scales larger than the cut-off frequency the dynamics is determined by white noise. Actually, when the *F*_*q*_*(n)* curves are estimated with maximally overlapped blocks, they run in parallel (see inset) with slope α close to 0.5 at the largest scale. In the example of a random series with multifractal Cauchy distribution (Figure 1, Lower), the large variability of the estimate without overlapping makes it difficult to identify the scale where the focus occurs and may completely hide possible crossover points between *n* = 10^3^ and *n* = 10^4^ for positive *q*. These drawbacks are largely mitigated by using maximum overlapping but at the cost of an increased computational load that may lead to unacceptable calculation times for long series.

To overcome this limit, we designed a fast algorithm for *F*_*q*_*(n)* calculation, particularly efficient in case of maximally overlapped blocks. Therefore, the aim of the present work is (1) to describe the theoretical aspects on which we based our fast algorithm; (2) to illustrate its performance by comparison with the traditional DFA algorithm; (3) to show potential applications in the fields of heart rate variability analysis and of EEG signals analysis; and (4) to provide the source code for its implementation. The algorithm optimizes the computation time of *F*_*q*_*(n)* and readers can then estimate α(*q,n*) with any of the methods proposed in literature: Kalman filtering, least-square regression over a running window or first derivative of log *F*_*q*_*(n)* vs. log *n*. In this work, we also include the code for slope estimation with the latter method because our fast algorithm was specifically designed for evaluating α(*q,n*) with the derivative approach.

## Fast *F_*q*_(n)* Calculation

Given a time series of *N* samples, *S*_*j*_, with *j* = 1, … *N*, mean μ_*S*_ and standard deviation σ_*S*_, the mean is removed and the series normalized to unit standard deviation:

(1) $$\begin{array}{l} s_{j}=\frac{S_{j}-\mu_{S}}{\sigma_{S}} \end{array}$$

The normalization in Equation (1), not required by DFA, is useful for recognizing data blocks with very low dynamics (see section *Calculation precision and small blocks overfitting*). The cumulative sum *y*_*i*_, with *i* = 1,… *N*, is:

(2) $$\begin{array}{l} y_{i}=\overset{i}{\underset{j=1}{\sum }}s_{j} \end{array}$$

To evaluate *F*_*q*_, the *y*_*i*_ series is split into consecutive blocks of size *n*. At a given scale *n*, the number of blocks, *M*, depends on the length of the series *N*, on the block size *n* and on the number of overlapped samples between consecutive blocks, *L*, with *0* ≤ *L*<*n*, as:

(3) $$\begin{array}{l} M=\left\lfloor \frac{N-n}{n-L}\right\rfloor +1 \end{array}$$

If *N-n* is not multiple of *n-L*, a short segment of *N-(M-1)(n-L)-n* samples at the end of the series are excluded from the analysis. In the case of maximum overlapping, when *L* = *n-1*, all the *N* samples contribute to the estimation of *F*_*q*_*(n)* and the number of blocks reaches the highest value: *M* = *N-n* + *1*. Each of the *M* blocks is detrended by least-square fitting a polynomial *p(i)*. The usual notation to indicate a DFA employing a polynomial of order *O* is DFA_*O*_. The variance of the residuals in the *k-th* detrended block is

(4a) $$\begin{array}{l} \sigma_{n}^{2}\left(k\right)=\frac{1}{n}\overset{I_{k}+n-1}{\underset{i=I_{k}}{\sum }}{\left(y_{i}-p\left(i\right)\right)}^{2} \end{array}$$

with *I*_*k*_ = (*n-L*)×(*k*-1)+1 index of the first sample falling into the *k-th* block. According to Kantelhardt et al. (2002), the variability functions for DFA multifractal analysis are:

(5) $$\begin{array}{l} \left\{ \begin{array}{l} F_{q}\left(n\right)={\left(\frac{1}{M}{\overset{M}{\underset{k=1}{\sum }}\left(\sigma_{n}^{2}\left(k\right)\right)}^{q/2}\right)}^{1/q}\text{for }q\neq \text{0}\\ F_{q}\left(n\right)=e^{\frac{1}{2M}\overset{M}{\underset{k=1}{\sum }}\ln\;\left(\sigma_{n}^{2}\left(k\right)\right)}\;\text{for }q=\text{0} \end{array}\right. \end{array}$$

Because of the normalization in Equation (1), the variability function of the original series is *F*_*q*_*(n)*×σ_*S*_.

Most of the computational load for evaluating *F*_*q*_*(n)* is due to the calculation of Equation (4a), which requires (1) a least-square polynomial fitting and (2) the evaluation of the variance of the residuals, over *n* points. These steps should be repeated for each of the *M* blocks and *M* reaches very large values, close to *N*, for maximally overlapped blocks (Equation 3). In the following, we describe how to make these calculation steps faster for detrending polynomials of order 1 or 2, i.e., for DFA_1_ and DFA_2_. We did not consider polynomials of a higher order because all the DFA biomedical applications we are aware of did not employ detrending polynomials of order greater than 2. However, the same optimization strategy we present here can be extended to higher orders.

### Least Square Polynomial Fitting

Freely available DFA codes, as accessible in Ihlen (2012) or described in Peng et al. (1995) and Gieraltowski et al. (2012) and downloadable in Goldberger et al. (2000), calculate the polynomial *p(i)* of order *O* with algorithms for least-square fitting a data-set of *n* points with coordinates (*i*,*y*_*i*_). This means solving *n* linear equations operating with a *n*×*(O*+*1)* Vandermonde matrix. The fitting polynomials of first and second order are:

(6a) $$\begin{array}{l} y=b_{1}i+a_{1} \end{array}$$

(6b) $$\begin{array}{l} y=c_{2}i^{2}+b_{2}i+a_{2} \end{array}$$

Coefficients of Equation (6a) are:

(7a) $$\begin{array}{l} b_{1}=\frac{S_{iy}}{S_{ii}} \end{array}$$

(7b) $$\begin{array}{l} a_{1}=\bar{y}-b_{1}\bar{i} \end{array}$$

with

(8a) $$\begin{array}{l} \bar{i}=\frac{1}{n}\overset{I_{k}+n-1}{\underset{i=I_{k}}{\sum }}i \end{array}$$

(8b) $$\begin{array}{l} \bar{y}=\frac{1}{n}\overset{I_{k}+n-1}{\underset{i=I_{k}}{\sum }}y_{i} \end{array}$$

(8c) $$\begin{array}{l} S_{iy}=\frac{1}{n}\overset{I_{k}+n-1}{\underset{i=I_{k}}{\sum }}iy_{i}-{\bar{i}}_{\bar{y}} \end{array}$$

(8d) $$\begin{array}{l} S_{ii}=\frac{1}{n}\overset{I_{k}+n-1}{\underset{i=I_{k}}{\sum }}i^{2}-{\bar{i}}^{2} \end{array}$$

Coefficients of Equation (6b) are:

(9a) $$\begin{array}{l} c_{2}=\frac{S_{i^{2}y}S_{ii}-S_{iy}S_{ii^{2}}}{S_{ii}S_{i^{2}i^{2}}-{\left(S_{ii^{2}}\right)}^{2}} \end{array}$$

(9b) $$\begin{array}{l} b_{2}=\frac{S_{iy}S_{i^{2}i^{2}}-S_{i^{2}y}S_{ii^{2}}}{S_{ii}S_{i^{2}i^{2}}-{\left(S_{ii^{2}}\right)}^{2}} \end{array}$$

(9c) $$\begin{array}{l} a_{2}=\bar{y}-b_{2}\bar{i}-c_{2}\bar{i^{2}} \end{array}$$

with, in addition to Equation (8a–d):

(10a) $$\begin{array}{l} \bar{i^{2}}=\frac{1}{n}\overset{I_{k}+n-1}{\underset{i=I_{k}}{\sum }}i^{2} \end{array}$$

(10b) $$\begin{array}{l} S_{ii^{2}}=\frac{1}{n}\overset{I_{k}+n-1}{\underset{i=I_{k}}{\sum }}i^{3}-\bar{i}\bar{i^{2}} \end{array}$$

(10c) $$\begin{array}{l} S_{i^{2}i^{2}}=\frac{1}{n}\overset{I_{k}+n-1}{\underset{i=I_{k}}{\sum }}i^{4}-\bar{i^{2}}\bar{i^{2}} \end{array}$$

(10d) $$\begin{array}{l} S_{i^{2}y}=\frac{1}{n}\overset{I_{k}+n-1}{\underset{i=I_{k}}{\sum }}i^{2}y_{i}-\bar{i^{2}}\bar{y} \end{array}$$

Equations (8a,d) and Equations (10a–c) require summing powers “*V”* of *i*, with *V* an integer between 1 and 4 and *i* consecutive natural numbers. Remembering the Faulhaber's formula:

(11) $$\begin{array}{l} \overset{n}{\underset{i=1}{\sum }}i^{V}=\frac{1}{V+1}\overset{V+1}{\underset{i=1}{\sum }}{\left(-1\right)}^{\delta_{iV}}\left(\begin{array}{c} V+1\\ i \end{array}\right)B_{V+1-i}n^{i} \end{array}$$

where *B*_*i*_ is the *i*-th Bernoulli's number, $\left(\begin{array}{c} V\\ i \end{array}\right)$ is the binomial coefficient, and δ_*iV*_ is the Kroenecker's delta (Weisstein, 1999), we have:

(12a) $$\begin{array}{l} \overset{n}{\underset{i=1}{\sum }}i=\frac{n^{2}}{2}+\frac{n}{2} \end{array}$$

(12b) $$\begin{array}{l} \overset{n}{\underset{i=1}{\sum }}i^{2}=\frac{n^{3}}{3}+\frac{n^{2}}{2}+\frac{n}{6} \end{array}$$

(12c) $$\begin{array}{l} \overset{n}{\underset{i=1}{\sum }}i^{3}=\frac{n^{4}}{4}+\frac{n^{3}}{2}+\frac{n^{2}}{4} \end{array}$$

(12d) $$\begin{array}{l} \overset{n}{\underset{i=1}{\sum }}i^{4}=\frac{n^{5}}{5}+\frac{n^{4}}{2}+\frac{n^{3}}{3}-\frac{n}{30} \end{array}$$

Since

(13) $$\begin{array}{l} \overset{I_{k}+n-1}{\underset{i=I_{k}}{\sum }}i^{V}=\overset{I_{k}+n-1}{\underset{i=1}{\sum }}i^{V}-\overset{I_{k}-1}{\underset{j=1}{\sum }}j^{V} \end{array}$$

the sum of powers of *n* consecutive integers does not require the actual summation of *n* terms and can be obtained through the much faster expressions of Equations (12). However, Equations (8b,c, 10d) include sums of the product between *i*^*V*^*y*_*i*_, with *V* integer between 0 and 2. There are no analytic expressions for these sums but a fast way to calculate them is to define the arrays *A*_*v, w*_(*i*), *i* = 0,…,*N*, as:

(14) $$\begin{array}{l} \left\{ \begin{array}{l} A_{V,W}\left(i\right)=0\;\text{ for }i=0\\ A_{V,W}\left(i\right)=A_{V,W}\left(i-1\right)+i^{V}y_{i}^{W}\text{ for }i>0 \end{array}\right. \end{array}$$

Once the arrays have been initialized, the sum of *n* consecutive *i*^*V*^$y_{i}^{W}$ products is calculated by the following difference:

(15) $$\begin{array}{l} \overset{I_{k}+n-1}{\underset{i=I_{k}}{\sum }}i^{V}y_{i}^{W}=A_{V,W}\left(I_{k}+n-1\right)-A_{V,W}\left(I_{k}-1\right) \end{array}$$

### Variance of the Residuals

To avoid another summation over *n* points, let's writing the squared expression in Equation (4a) for the linear fitting (Equation 6a) as:

(16a) $$\begin{array}{l} \sigma_{n}^{2}\left(k\right)=\frac{1}{n}\overset{I_{k}+n-1}{\underset{i=I_{k}}{\sum }}{\left[y_{i}-\left(b_{1}i+a_{1}\right)\right]}^{2}=\\ =a_{1}^{2}+\frac{1}{n}[\overset{I_{k}+n-1}{\underset{i=I_{k}}{\sum }}{y_{i}}^{2}+{b_{1}}^{2}\overset{I_{k}+n-1}{\underset{i=I_{k}}{\sum }}i^{2}\\ +2a_{1}b_{1}\overset{I_{k}+n-1}{\underset{i=I_{k}}{\sum }}i-2b_{1}\overset{I_{k}+n-1}{\underset{i=I_{k}}{\sum }}iy_{i}-2a_{1}\overset{I_{k}+n-1}{\underset{i=I_{k}}{\sum }}y_{i}] \end{array}$$

and for quadratic fitting (Equation 6b) as:

(16b) $$\begin{array}{l} \sigma_{n}^{2}\left(k\right)=\frac{1}{n}\overset{I_{k}+n-1}{\underset{i=I_{k}}{\sum }}{\left[y_{i}-\left(c_{2}i^{2}+b_{2}i+a_{2}\right)\right]}^{2}=\\ =a_{2}^{2}+\frac{1}{n}[{c_{2}}^{2}\overset{I_{k}+n-1}{\underset{i=I_{k}}{\sum }}i^{4}+2c_{2}b_{2}\overset{I_{k}+n-1}{\underset{i=I_{k}}{\sum }}i^{3}+\left(b_{2}^{2}+2a_{2}c_{2}\right)\overset{I_{k}+n-1}{\underset{i=I_{k}}{\sum }}i^{2}\\ +2b_{2}a_{2}\overset{I_{k}+n-1}{\underset{i=I_{k}}{\sum }}i]+\\ +\frac{1}{n}\left[\overset{I_{k}+n-1}{\underset{i=I_{k}}{\sum }}{y_{i}}^{2}-2c_{2}\overset{I_{k}+n-1}{\underset{i=I_{k}}{\sum }}i^{2}y_{i}-2b_{2}\overset{I_{k}+n-1}{\underset{i=I_{k}}{\sum }}iy_{i}-2a_{2}\overset{I_{k}+n-1}{\underset{i=I_{k}}{\sum }}y_{i}\right] \end{array}$$

The sums in Equations (16a,b) are obtained analytically with Equations (12a–d, 13), and directly from Equation (15) with *V* = 0 and *W* = 1 or 2, and with *W* = 1 and *V* = 1 or 2.

## Calculation Precision

To avoid subtracting terms with very different magnitude, which may introduce errors due to the finite precision of numbers representation, the starting point of the *k*-th block, *I*_*k*_, is shifted to 1 rewriting Equation (4a) as:

(4b) $$\begin{array}{l} \sigma_{n}^{2}\left(k\right)=\frac{1}{n}\overset{n}{\underset{j=1}{\sum }}{\left(y_{j+I_{k}-1}-p^{\prime }\left(j\right)\right)}^{2} \end{array}$$

The difference in Equation (13) is no more required and Equation (15) is calculated as in Appendix. However, numerical errors may affect *A*_*v,w*_(*i*) in Equation (14). In fact, the array initialization requires adding the term *i*^*V*^$y_{i}^{W}$ to a running summation *A*_*V, W*_(*i-1*) which may reach very high values for long series when *i* is close to *N*, propagating precision errors from the lower to the higher indices *i*. To avoid this problem, the running summation is split into a sequence of shorter sums over segments of 128 samples. This is done defining the matrix **A**_*V, W*_(*r,c*) of 129 rows (0 ≤ *r* ≤ 128) and *Q* = ⌊(*N*-1)/128⌋+1 columns (1 ≤ *c* ≤ *Q*), as:

(17) $$\begin{array}{l} \left\{ \begin{array}{l} {\text{A}}_{V,W}\left(r,c\right)=0\;\text{for }r=0\\ {\text{A}}_{V,W}\left(r,c\right)={\text{A}}_{V,W}\left(r-1,c\right)\\ \;+{\left[r+\left(c-1\right)\times 128\right]}^{V}y_{r+\left(c-1\right)\times 128}^{W}\text{ for }r>0 \end{array}\right. \end{array}$$

Any index *i* > 0 corresponds to the column *c* = ⌊(*i*-1)/128⌋+1 and to the row *r* = *i*-*c*×128, and *A*_*V,W*_(*i*) is calculated as

(18) $$\begin{array}{l} \left\{ \begin{array}{l} A_{V,W}\left(i\right)={\text{A}}_{V,W}\left(r,c\right)\text{ for }c=1\\ A_{V,W}\left(i\right)={\text{A}}_{V,W}\left(r,c\right)+\overset{c-1}{\underset{k=1}{\sum }}{\text{A}}_{V,W}\left(128,k\right)\text{ for }c>1 \end{array}\right. \end{array}$$

avoiding most of the sums that include terms with very different magnitude.

For extremely long series, even Equation (18) cannot exclude errors due to the finite precision of numbers representation. To control further these errors, the variance of the residuals in Equation (4b) is compared with a threshold. The idea is that when the variance of the residuals is extremely low, the relative weight of errors due to the finite precision of numbers representation might have a detectable influence on the estimate. In this case, the variance is recalculated *directly* as the summation of the *n* consecutive powers *i*^*V*^$y_{i}^{W}$ and not through (Equation 15). Because of the normalization in Equation (1), the threshold does not depend on the standard deviation of the time series, but only on its length *N*, on the precision of number representation and on the detrending order. In our implementation the default value of the DFA_1_ threshold, *Th*_1_, is

(19a) $$\begin{array}{l} \left\{ \begin{array}{l} Th_{1}=10^{-3}\;\text{for }N\leq 10^{5}\\ Th_{1}=\frac{N}{10^{8}}\;\text{for }N>10^{5} \end{array}\right. \end{array}$$

and of the DFA_2_ threshold, *Th*_2_, is

(19b) $$\begin{array}{l} \left\{ \begin{array}{l} Th_{2}=10^{-2}\;\text{for }N\leq 10^{2}\\ Th_{2}=\frac{N}{10^{4}}\;\text{for }N>10^{2} \end{array}\right. \end{array}$$

A numerical problem of different nature may require comparing the variance of the residuals in Equation (4b) with another threshold *EPS*, as described in Ihlen (2012). A detrending polynomial of too high order might overfit the blocks of smaller size *n*, or pre-processing procedures, as low-pass filtering, might remove fractal components at the shortest scales. In these cases, the variance of the residuals could be so low to severely distort log *F*_*q*_*(n)* at negative *q*. The solution proposed in Ihlen (2012) is to discard variances lower than an *EPS* threshold set “*to the precision of the measurement device that is recording the biomedical time series*.” Since we normalized the series to unit variance, our approach is to set *EPS* as a fraction of the dynamics of the original series (for instance, EPS = 10^−4^).

## Algorithm Performance

To assess numerical stability and speed of our algorithm (see Matlab source code in the Supplemental File *FMFDFA.m*), we applied it to synthesized time series. We synthesized three series of *N* = 10^6^ samples: a white Gaussian noise with zero mean and unit variance, {wn_*i*_}; a Brownian motion as the integral of a zero-mean white noise with variance = 0.01986918, {Bm_*i*_}; and the series {wb_*i*_} obtained as their superposition, i.e.,:

(20) $$\begin{array}{l} wb_{i}\;=wn_{i}\;+\;Bm_{i} \end{array}$$

**Figure 2:**
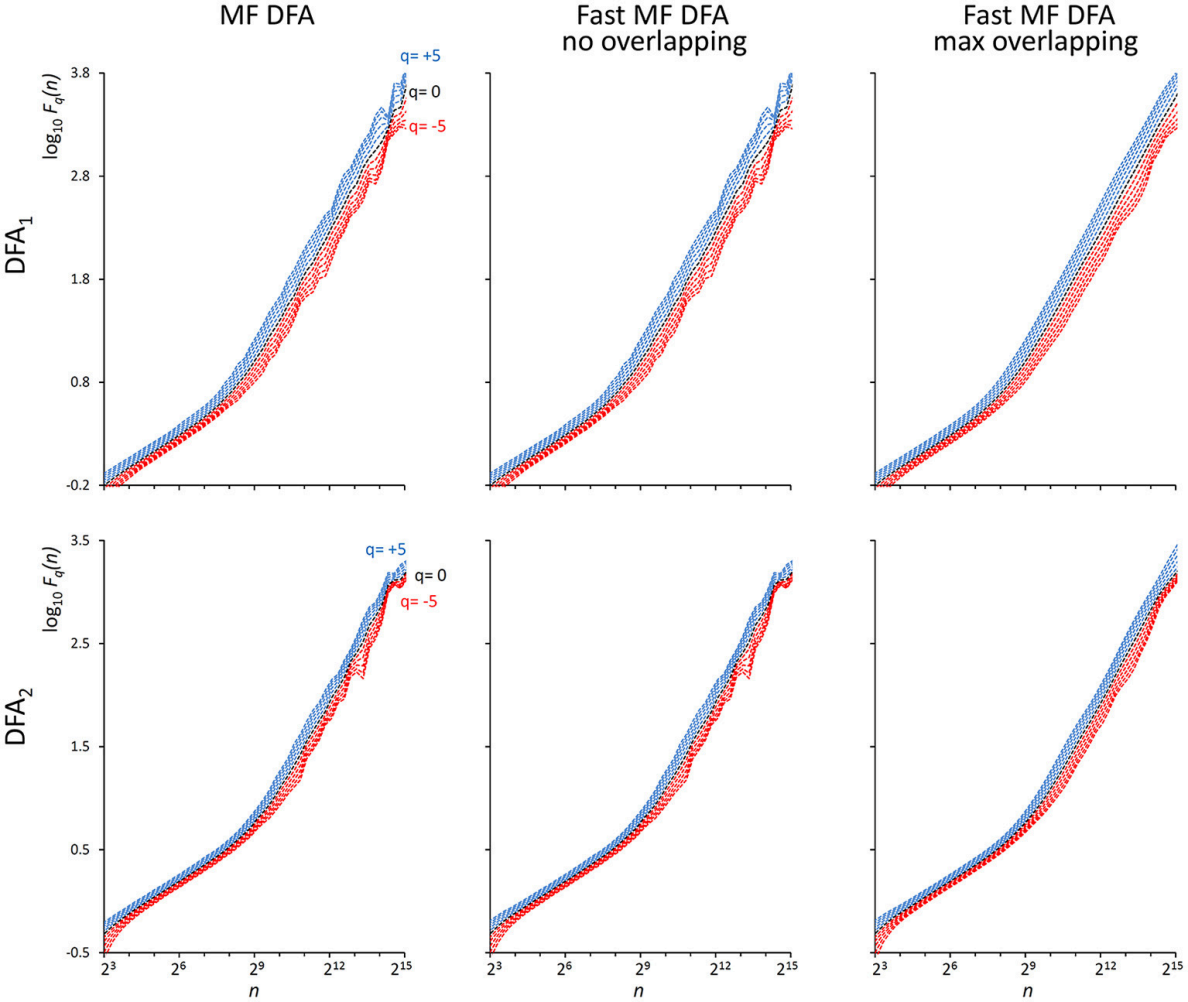
*F*_*q*_*(n)* functions by the traditional (MF DFA) and the proposed fast algorithm (Fast MF DFA) for the {wb_*i*_} series. The series of *N* = 200,000 samples is the superposition of white noise and Brownian motion (see text); estimates without overlapping by the traditional algorithm **(Left)** and by our fast algorithm **(Center)** and with maximum overlapping by our fast algorithm **(Right)** were performed over 56 block sizes exponentially distributed over the *n* axis, with thresholds *Th*_1_ and *Th*_2_ as in Equations (19) and *EPS* = 0. On a laptop computer, the traditional algorithm employed T = 57 s for calculating DFA_1_ and T = 146 s for calculating DFA_2_ without overlapping, the fast algorithm employed 2.2 s without overlapping and 65 s with maximum overlapping for calculating both the DFA_1_ and DFA_2_ estimates simultaneously.

As indicated in Kiyono (2015), the power spectrum of {wb_i_} has a crossover frequency separating the spectral profiles of white and brown noises at *f*_*co*_ = 10^−2.5^, corresponding to a crossover scale *n*_*co*_ = 316 samples. Figure 2 shows an example of DFA_1_ and DFA_2_ estimates on *N* = 200,000 samples of {wb_i_} (we analyze EEG series of the same length in the next paragraph “*Application on real biomedical time series”*).

To evaluate how the finite precision of number representation may affect the sum of *n* consecutive products when obtained by Equation (15), we extracted segments of length *N* = 10^*k*+1^, with *k* integer between 1 and 5, from the three synthesized series. For each segment, we estimated *F*_*q*_*(n)* for all integers *q* between −5 and +5 and for scales *n*_*b*_ = 10^*b*^ with *b* an integer between 1 and *k*-1 (e.g., for a segment of length *N*_4_ = 10,000 samples we considered the scales *n*_1_ = 10, *n*_2_ = 100 and *n*_3_ = 1,000 samples). Estimates were performed with the fast algorithm with maximally overlapped blocks, setting *Th*_1_ and *Th*_2_ as in Equations (19) and EPS = 0. These “fast” estimates were compared with estimates from a reference algorithm in which the difference on the right side of Equation (15) is replaced by the slower but more precise summation of *n* products on the left side of the equation. The relative error between the fast estimate, *F*_*q*_*(n*_*b*_*)*^*F*^, and the reference estimate, *F*_*q*_*(n*_*b*_*)*^*R*^, was computed for each *q* and each block size *n*_*b*_ as:

(21) $$\begin{array}{l} \varepsilon \left(q,n_{b}\right)=|F_{q}{\left(n_{b}\right)}^{F}-F_{q}{\left(n_{b}\right)}^{R}|/F_{q}{\left(n_{b}\right)}^{R} \end{array}$$

The largest of these errors among all the *n*_*b*_ block sizes, among all the *q* values and among the three series ({wb_*i*_}, {wn_*i*_} and {Bm_*i*_}) was taken as a global measure of the precision of the fast algorithm for any given length *N*_k_. Relative errors are reported in Table 1: even in the worst case (corresponding to the length *N* = 10^6^ samples), the highest relative error is less than 1%.

**Table 1:** Largest relative error in *F*_*q*_*(n)* estimates induced by calculating the sum of *n* products with the fast Equation (15).

	**Time series**				
	**{wn**_**i**_**}; {Bm**_**i**_**}; {wb**_**i**_**};**			**{bcn**_**i**_**};**	
**Length**	**DFA**_**1**_	**DFA**_**2**_	**Length**	**DFA**_**1**_	**DFA**_**2**_
*N* = 10^2^	1.4E-11	1.9E-11	*N* = 2^8^	3.1E-11	2.1E-11
*N* = 10^3^	1.0E-09	2.3E-08	*N* = 2^10^	4.8E-10	1.2E-10
*N* = 10^4^	1.0E-07	5.0E-07	*N* = 2^12^	2.6E-09	3.9E-09
*N* = 10^5^	1.9E-05	6.1E-06	*N* = 2^14^	1.1E-06	4.0E-07
*N* = 10^6^	6.0E-03	4.4E-04	*N* = 2^16^	4.6E-06	2.2E-06

*{wn_i_}, white noise; {Bm_i_}, Brownian motion; {wb_i_}, sum of {wn_i_} and {Bm_i_}; {bcn_i_}, stochastic binomial cascade embedded in noise (see text for details)*.

**Figure 3:**
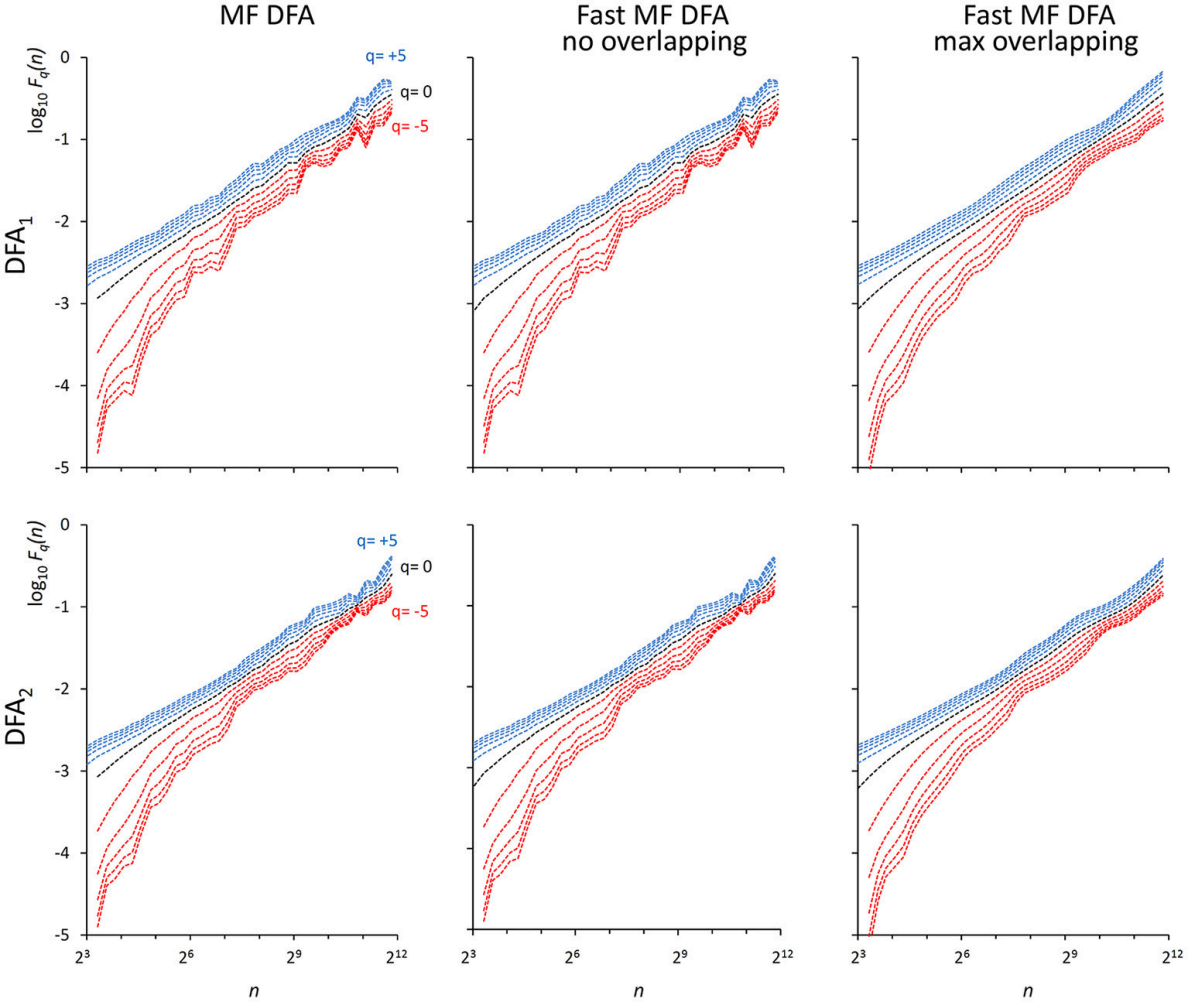
*F*_*q*_*(n)* functions for a multifractal binomial cascade embedded in noise of *N* = 65,536 samples. Estimates without overlapping by the traditional **(Left)** and fast **(Center)** algorithm and with maximum overlapping by the fast algorithm **(Right)** were performed over 38 block sizes, with thresholds as in Equations (19) and *EPS* = 0. On a laptop computer, the traditional algorithm employed *T* = 4.3 s for DFA_1_ and *T* = 9.5 s for DFA_2_ without overlapping while the fast algorithm employed 0.12 s without overlapping and 3.6 s with maximum overlapping for calculating DFA_1_ and DFA_2_ estimates simultaneously.

We also generated a stochastic binomial cascade embedded in noise, {bcn_i_}, with a procedure similar to that described in Gieraltowski et al. (2012). We started from a series of *N* = 2^14^ samples equal to 1, we split it into two segments of the same length, and multiplied one segment by the weight 0.25 and one by the weight (1-0.25), randomly selecting the weights of the first and second segment. We repeated the splitting/weighting procedure for each segment of the previous step, up to 14 steps. To include random noise, we substituted all values lower than 10^−6^ with random samples uniformly distributed between 0 and 0.01. From this cascade, we subtracted its reversed duplicate to obtain a symmetric distribution. Figure 3 shows examples of *F(n)* estimates for the {bcn_i_} series (we analyzed heart-rate series of similar length in the paragraph “*Application on real biomedical time series”*). We generated and concatenated four of these binomial cascades to obtain a series of length *N* = 2^16^. Relative errors were assessed from fast, *F*_*q*_*(n*_*b*_*)*^*F*^, and reference, *F*_*q*_*(n*_*b*_*)*^*R*^, estimates (calculated with maximally overlapped blocks, thresholds as in Equation 19 and EPS = 0) for segments of length *N*_*k*_ = 2^2*k*+6^ with 1 ≤ *k* ≤ 5, and block sizes *n*_*b*_ = 2^2*b*+1^ with 1 ≤ *b* ≤ *k* (e.g., for the length *N*_4_ = 16,384 we considered the scales *n*_1_ = 16, *n*_2_ = 64, *n*_3_ = 256 and *n*_4_ = 1,024). Results in Table 1 show negligible errors also for this multifractal series.

**Figure 4:**
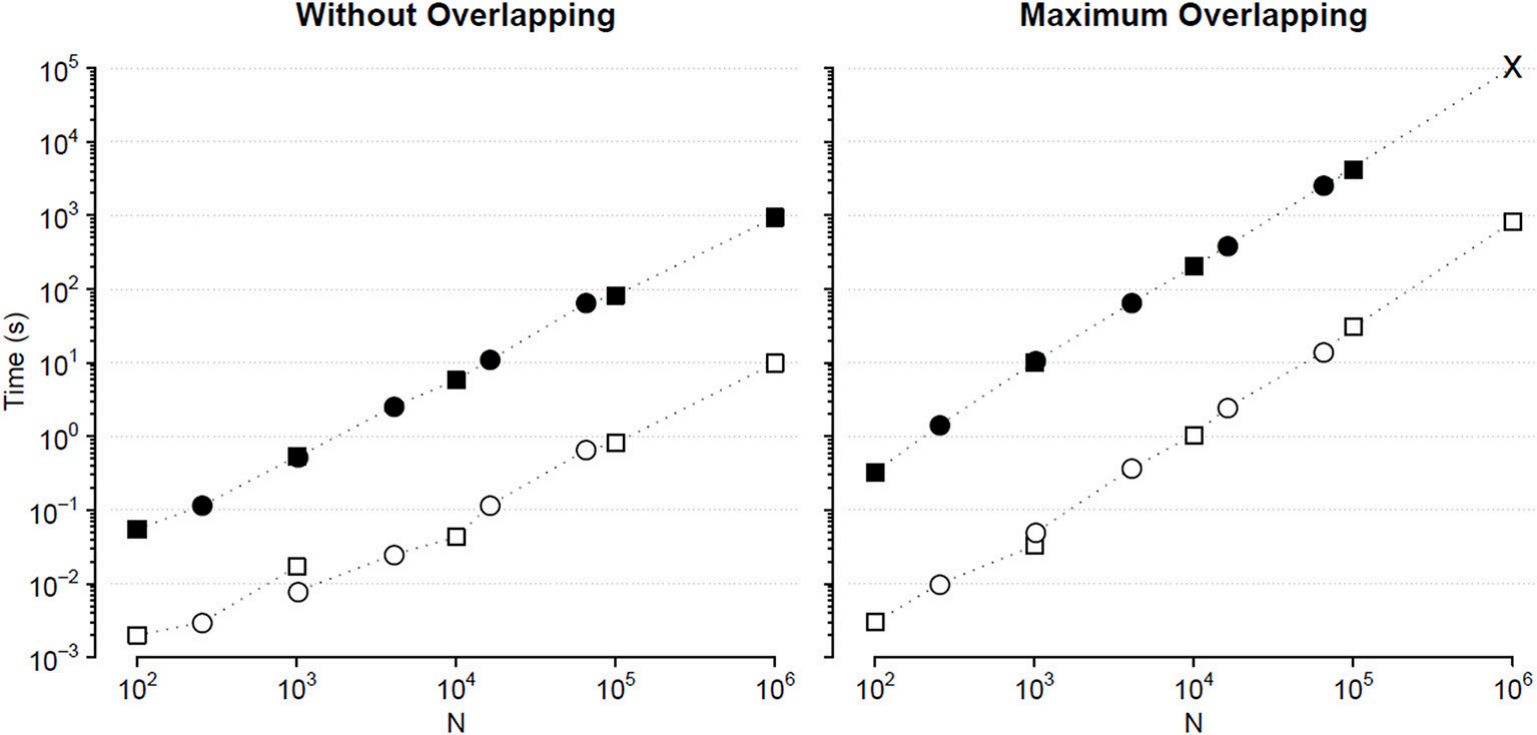
Comparison of *F*_*q*_*(n)* calculation times (sum of DFA_1_ and DFA_2_) for series of length *N*. Times for analyzing {wb_i_} (square) and binomial cascade (circle) series (see text) by traditional (solid symbols) and fast MF DFA algorithm (open symbols): average of 10 runs on a personal computer with Intel i7-7600U CPU at 2.80 GHz, 16 GB RAM and Samsung MZVLW1T0HMLH-000L7 hard disk. As the series length, *N*, increases, the number of block sizes, *n*, also increases remaining constant the block-size density of four *n* values per decade. The calculation time of the traditional algorithm for maximum overlapping and *N* = 10^6^ has been extrapolated (cross symbol).

We compared the calculation times employed by the traditional and the fast MF DFA algorithm to analyze {wb_i_} series of length *N*_*k*_ = 10^*k*+1^ and binomial cascade series of length *N*_*k*_ = 2^2*k*+6^, with 1 ≤ *k* ≤ 5. Results in Figure 4 indicate that the fast algorithm is about two orders of magnitude faster than the traditional one.

## Local Slopes of *F_*q*_(n)*

Once that *F*_*q*_*(n)* has been estimated with maximally overlapped blocks, the multifractal DFA coefficients α(*q,n*) may be obtained as the first derivative of log *F*_*q*_*(n)* vs. log *n*. To have equispaced log *n* values we interpolate the log *F*_*q*_*(n)* samples by a spline function, obtaining *H* values *F*_*q*_*(n*_*h*_*)* with *n*_*h*_ real numbers spaced exponentially on the scale axis. The slope at *n*_*h*_ is the derivative formula on 5 points (Castiglioni et al., 2018):

(22a) $$\begin{array}{l} \alpha \left(q,n_{h}\right)\\ =\frac{8\left(\log F_{q}\left(n_{h+1}\right)-\log F_{q}\left(n_{h-1}\right)\right)-\left(\log F_{q}\left(n_{h+2}\right)-\log F_{q}\left(n_{h-2}\right)\right)}{3\left(\log\left(n_{h+2}\right)-\log\left(n_{h-2}\right)\right)}\\ \text{for }2<h<H-1 \end{array}$$

or on 3 points at the extremes of the scale axis:

(22b) $$\begin{array}{l} \alpha \left(q,n_{h}\right)=\frac{-\log F_{q}\left(n_{h+2}\right)+4\log F_{q}\left(n_{h+1}\right)-3\log F_{q}\left(n_{h}\right)}{\log\left(n_{h+2}\right)-\log\left(n_{h}\right)}\text{ for }h=1 \end{array}$$

(22c) $$\begin{array}{l} \alpha \left(q,n_{h}\right)=\frac{\log F_{q}\left(n_{h+1}\right)-\log F_{q}\left(n_{h-1}\right)}{\log\left(n_{h+1}\right)-\log\left(n_{h-1}\right)}\;\text{for }h=2\text{ and }h=H-1 \end{array}$$

(22d) $$\begin{array}{l} \alpha \left(q,n_{h}\right)=\frac{\log F_{q}\left(n_{h-2}\right)-4\log F_{q}\left(n_{h-1}\right)+3\log F_{q}\left(n_{h}\right)}{\log\left(n_{h}\right)-\log\left(n_{h-2}\right)}\text{ for }h=H \end{array}$$

The code for calculating Equations (22) is available as the Supplemental Matlab File *slpMFMSDFA.m*.

## Combining Information From DFA_1_ and DFA_2_

Our algorithm provides both DFA_1_ and DFA_2_ estimates in a single run. In some cases, there are reasons to prefer one of the two estimates. For instance, DFA_1_ may identify the scale where possible crossovers occur better than DFA_2_ (Höll and Kantz, 2015; Kiyono, 2015); however, if the power spectrum of the time series depends on the frequency *f* proportionally to 1/*f* ^β^, then DFA_2_ estimates the correct α up to β < 5, while DFA_1_ can estimate it only up to β < 3 (Kiyono, 2015). Furthermore, it is possible that the nature of the physiological system under analysis may make preferable one of the two detrending orders. For instance, in the field of heart-rate variability analysis, some studies highlighted that the slope α at *q* = 2 reflects the sympatho/vagal balance over scales shorter than 11 beats and allows stratifying the cardiovascular risk (Perkiomaki, 2011). Since high-order detrending polynomials overfit the shortest blocks (Kantelhardt et al., 2001), most of the clinical studies estimated this index by DFA_1_, and a first-order detrending should be used to get an autonomic index comparable with the medical literature. On the other hand, a crossover at the larger heart-rate scales was found for DFA_1_ but not for DFA_2_ during light sleep, suggesting that in this sleep phase DFA_2_ can better remove long-term trends (Bunde et al., 2000). This would make DFA_2_ preferable to DFA_1_ when comparing heart rate variability by phases of sleep over the larger scales.

**Figure 5:**
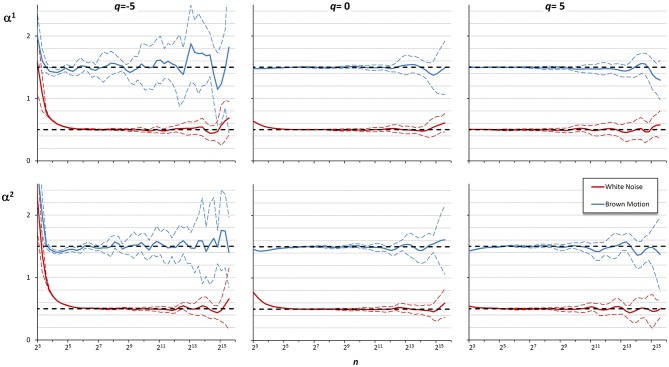
Multifractal multiscale coefficients by DFA_1_, α^1^, and by DFA_2_, α^2^. Values as derivative of log *F*_*q*_*(n)* vs. log *n* with maximally overlapped blocks: mean ± SD for 10 white noises and 10 Brownian motions. Dashed horizontal lines represent the theoretical value for white noise (=0.5) or Brownian motion (=1.5).

These examples suggest that the best detrending order may differ if the focus of the analysis is on the shorter rather than on the larger scales. For a multifractal series, it is even possible that the best detrending order may depend also on *q* because components with different amplitude may have different fractal structures. Therefore, it is conceivable that the assessment of α(*q,n*) could be improved by properly combining the results of DFA_1_ and DFA_2_ at each scale. To investigate this possibility, we considered 10 white-noise and 10 Brownian-motion series, each of *N* = 200,000 samples. Often the fractal dynamics of physiological time series is described as belonging to the family of fractional Gaussian noises and fractional Brownian motions (Eke et al., 2000), and the behavior at the extremes of this family of random processes (i.e., white noise and Brownian motion) may be assumed to represent a large class of physiological series. Then we calculated α(*q,n*) by DFA_1_ and by DFA_2_ with *q* = −5, *q* = 0, and *q* = +5, for each series. Figure 5 illustrates the deviations of the scale exponents from the theoretical value showing mean and standard deviation of the estimates separately over the groups of white noise and Brownian motion. The figure shows that at the shorter scales the estimation bias is greater for DFA_2_ while, at the larger scales, DFA_2_ appears more stable than DFA_1_ for *q* = −5, less stable than DFA_1_ for *q* = +5 and of quality similar to DFA_1_ for *q* = 0.

These findings suggest that if the time series is composed by fractional Gaussian noises and/or fractional Brownian motions, DFA_1_ is preferable to DFA_2_ at the shorter scales, while at the larger scales the choice may depend on the sign of *q*, and if *q* = 0 averaging the DFA_1_ and DFA_2_ coefficients might improve the estimate. Accordingly, we combined the estimates by DFA_1_, α^1^(*q,n*), and by DFA_2_, α^2^(*q,n*), defining a weighted average, α^w^(*q,n*), when *q* = −5, as:

(23a) $$\begin{array}{l} \left\{ \begin{array}{l} \alpha^{w}\left(-5,n\right)=\alpha^{1}\left(-5,n\right)\;\text{for }n<12\\ \alpha^{w}\left(-5,n\right)=\frac{24-n}{12}\alpha^{1}\left(-5,n\right)+\frac{n-12}{12}\alpha^{2}\left(-5,n\right)\text{ for }12\leq n\leq 24\\ \alpha^{w}\left(-5,n\right)=\alpha^{2}\left(-5,n\right)\;\text{for }n>24 \end{array}\right. \end{array}$$

when *q* = 0, as:

(23b) $$\begin{array}{l} \left\{ \begin{array}{l} \alpha^{w}\left(0,n\right)=\alpha^{1}\left(0,n\right)\;\text{for }n<12\\ \alpha^{w}\left(0,n\right)=\frac{36-n}{24}\alpha^{1}\left(0,n\right)+\frac{n-12}{24}\alpha^{2}\left(0,n\right)\;\text{for }12\leq n\leq 24\\ \alpha^{w}\left(0,n\right)=\frac{\alpha^{1}\left(0,n\right)+\alpha^{2}\left(0,n\right)}{2}\;\text{for }n>24 \end{array}\right. \end{array}$$

and when *q* = +5, as:

(23c) $$\begin{array}{l} \alpha^{w}\left(5,n\right)=\alpha^{1}\left(5,n\right) \end{array}$$

**Figure 6:**
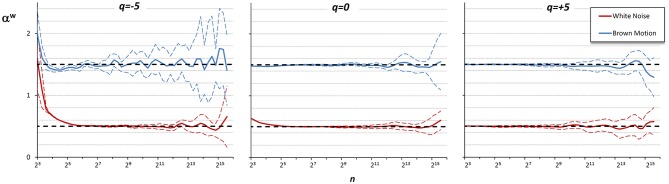
Multifractal Multiscale coefficients by combining DFA_1_ and DFA_2_. Weights defined by Equations (23a–c).

Figure 6 shows the α^w^ coefficients derived from α^1^ and α^2^ of Figure 5.

**Figure 7:**
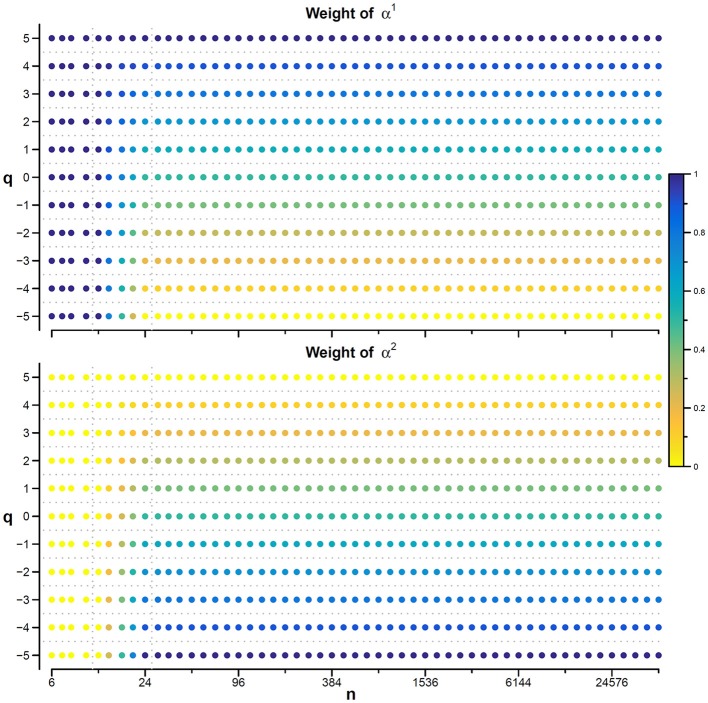
Weights for combining DFA_1_ and DFA_2_ estimates. The weighted estimate, α^w^(*q,n*), is a linear combination of DFA_1_ coefficients, α^1^(*q,n*), and DFA_2_ coefficients, α^2^(*q,n*), and it is proposed if there are no specific reasons to prefer one of the two detrending orders and if the time-series dynamics is assumed to be composed by fractional Gaussian noises or fractional Brownian motions. The weights, based on Equations (23), range between 0 (yellow) and 1 (dark blue). When *n* ≤ 12, α^w^ = α^1^ for all *q* values; when *n*≥24, α^w^ = α^1^ for *q* = 5, α^w^ = α^2^ for *q* = −5, and α^1^ and α^2^ weights change linearly with *q* between −5 and +5 so that α^w^ is the average between α^1^ and α^2^ for *q* = 0. The α^1^ and α^2^ weights change linearly also with *n* between 12 and 24.

Values of α^w^ for *q* between −5 and +5 are calculated linearly interpolating the weights for *q* = −5 and *q* = +5. Figure 7 illustrates how the weights for the mixed coefficients are defined.

## Applications on Real Biomedical Time Series

This section presents examples of applications of our algorithm on physiological time series collected in volunteers that gave their written informed consent to participate to the study, which was approved by the Ethics Committee of Istituto Auxologico Italiano, IRCCS, Milan, Italy.

### Heart Rate

Most of DFA applications in physiology regard the analysis of heart rate variability. In this field, the evaluation of the self-similarity exponents as a continuous function of the scale, α(*n*), proved useful for associating a short-term crossover to the dynamics of removal of noradrenaline released by the sympathetic nerve endings (Castiglioni, 2011), for quantifying alterations during sleep at high-altitude (Castiglioni et al., 2011b) and for evaluating clinical conditions like congestive heart failure (Bojorges-Valdez et al., 2007) or spinal lesions (Castiglioni and Merati, 2017). When the self-similarity exponents were estimated as a multifractal multiscale surface of the moment *q* and scale *n*, α(*q,n*), they provided information on the autonomic development from fetal heart rate recordings (Gieraltowski et al., 2013), on differences between the dynamics of heart rate and other cardiovascular variables (Castiglioni et al., 2018) and helped modeling the heart rate dynamics during sleep (Solinski et al., 2016).

**Figure 8:**
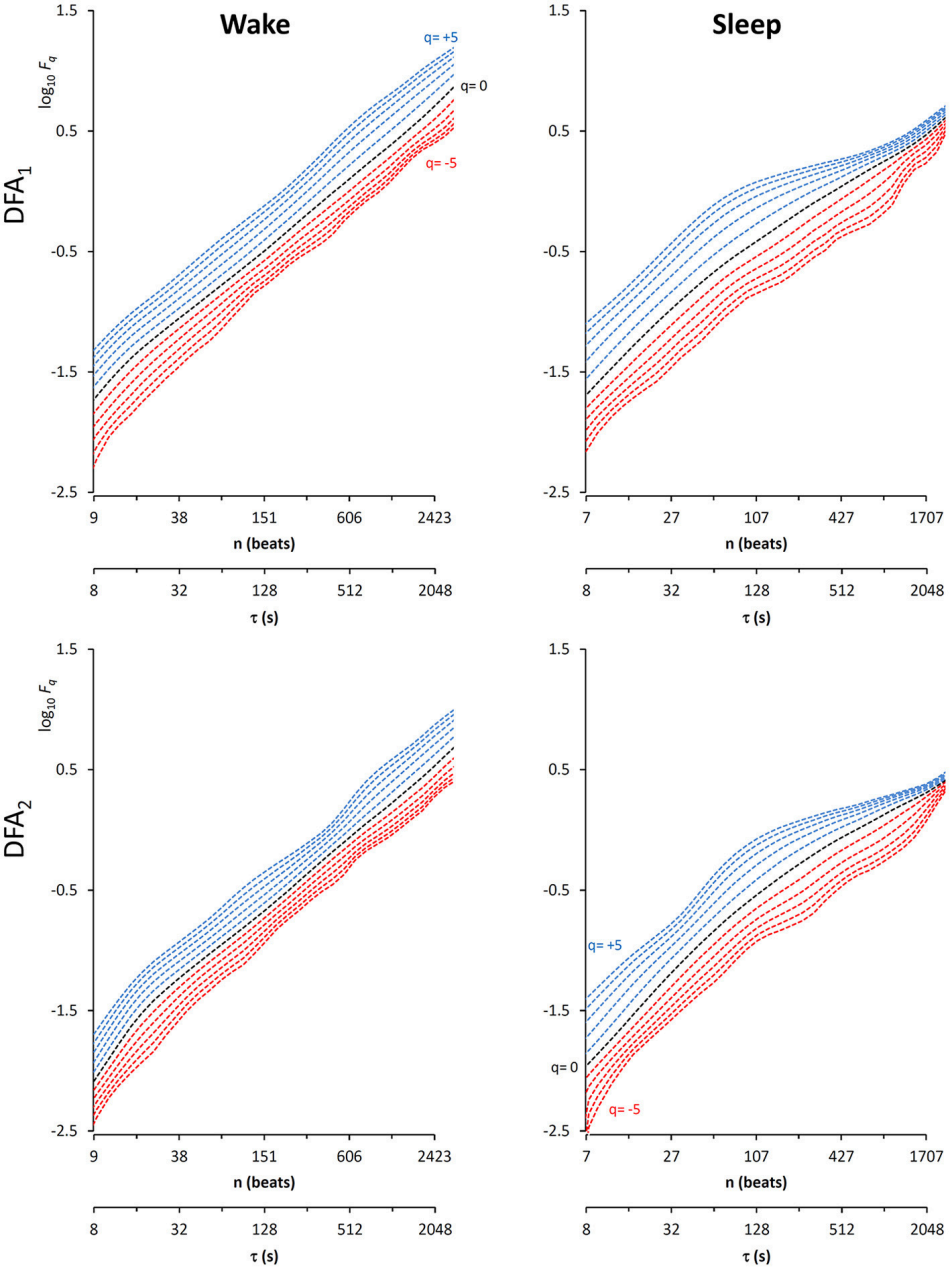
Multifractal fluctuation functions of heart-rate variability during Wake and Sleep with maximally overlapped blocks. The original series are two 4-h segments of beat-by-beat R-R intervals from a 24-h ECG recording in a healthy volunteer. The first horizontal axis is the box size *n*, in number of beats; the second horizontal axis is the temporal scale τ, in seconds, obtained multiplying *n* by the mean R-R interval, which is shorter in *Wake* than in *Sleep*. Thresholds *Th*_1_ and *Th*_2_ as in Equations (19) and *EPS* = 0.

**Figure 9:**
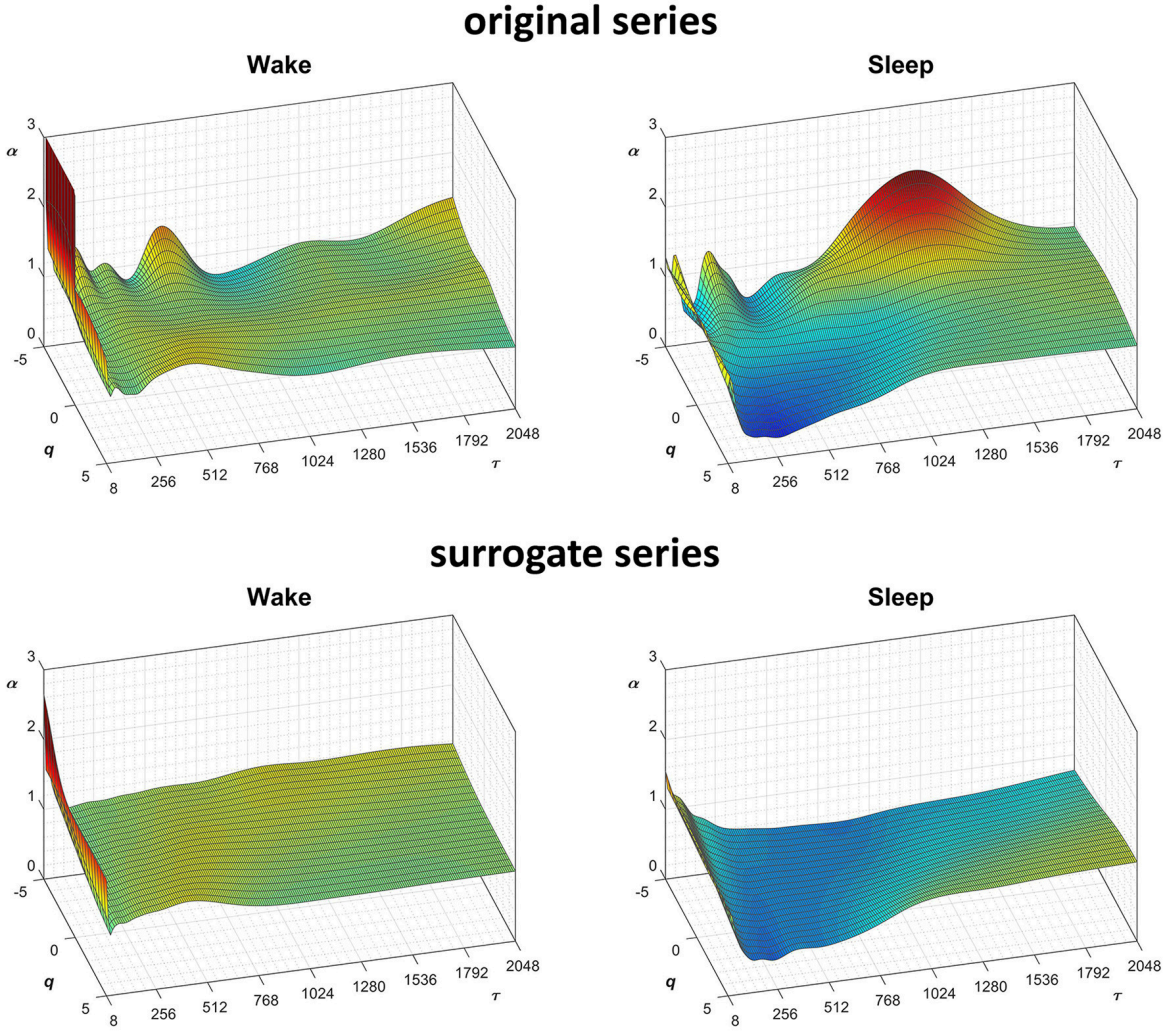
Scale coefficients of heart-rate variability during Wake and Sleep, for original and surrogate series. Coefficients calculated by Equations (22), combining DFA_1_ and DFA_2_ estimates by Equations (23); for surrogate data, the figure shows the average α surface over 100 series generated shuffling the phases of the Fourier spectrum of the original series. To more easily compare Wake and Sleep conditions, which are characterized by different heart rates, the τ axis represents the temporal scale in seconds; τ is obtained multiplying the box size, *n*, with the mean R-R interval (see Figure 8).

In this context, our fast algorithm is expected to improve existing methods by calculating α(*q,n*) surfaces with higher scale resolution and lower estimation variability, thanks to its ability to provide estimates with maximally overlapped blocks in short time. As an example in the field of heart rate variability, we compare multiscale multifractality during nighttime sleep and during daytime activities in a healthy male volunteer. We derived R-R interval series from a 24-h electrocardiogram sampled at 250 Hz. We compared *F*_*q*_*(n)* functions calculated on two segments of 4 h duration, one selected during daytime, from 2 p.m. to 6 p.m. (Wake condition), one during nighttime after sleep onset, from 2 a.m. to 6 a.m. (Sleep condition). The mean heart rate was 50 bpm during Sleep (*N* = 12,000 beats) and 71 bpm during Wake (*N* = 17,000 beats). Four premature beats were identified visually and removed, and *F*_*q*_*(n)* functions were derived by DFA_1_ and DFA_2_ with maximally overlapped blocks. Since the mean heart rate is lower in Sleep than in Wake condition, the same block of *n* beats corresponds to different time scales, in seconds, during Sleep and during Wake. Therefore, the fluctuation functions were plotted vs. the corresponding temporal scale τ, in seconds, by multiplying *n* by the mean R-R interval. Figure 8 shows that Wake and Sleep have different fluctuations functions, increasing with τ as parallel lines in Wake and converging to a focus at the longest scales in Sleep. This suggests a multifractal behavior (i.e., different slopes for different *q*), more evident at some scales (i.e., a multiscale dynamics), during sleep. The *F*_*q*_*(*τ*)* functions by DFA_1_ and by DFA_2_ appear similar: this confirms a previous observation on 24-h heart rate recordings (Gieraltowski et al., 2012) and does not suggest the presence of long-term trends that make one of the two detrending orders preferable. Therefore, we derived the surface of multifractal multiscale coefficients by applying the weighted approach of Equations (23). Figure 9 illustrates the resulting α(*q*,τ) surfaces. During daytime, the surface represents a relatively monofractal process with multiscale dynamics, i.e., α depends more on τ than on *q*. By contrast, during sleep, the surface also shows a strong dependence on *q* at some scales, like at τ = 1250 s, where it ranges between α = 2.05 at q = −5 and α = 0.9 at q = +5.

Fractal structures in physiological systems may arise from nonlinear chaotic dynamics or may be due to linear spectral features. We tested whether the α(*q,n*) surfaces we estimated for the heart-rate series actually reflect an underlying nonlinear dynamics with the method of surrogate data analysis (Theiler et al., 1992). For this purpose, we generated a dataset of 100 Fourier-phase shuffled time series (Schreiber and Schmitz, 2000) from each of the two recordings. The surrogate series have the same power spectrum of the original recording but a random phase. We calculated α(*q,n*) for the “Wake” and “Sleep” surrogate datasets. The average of each dataset is plotted in Figure 9 for comparison with the original data. Multifractal structures evident in the original series are absent in the surrogate data.

**Figure 10:**
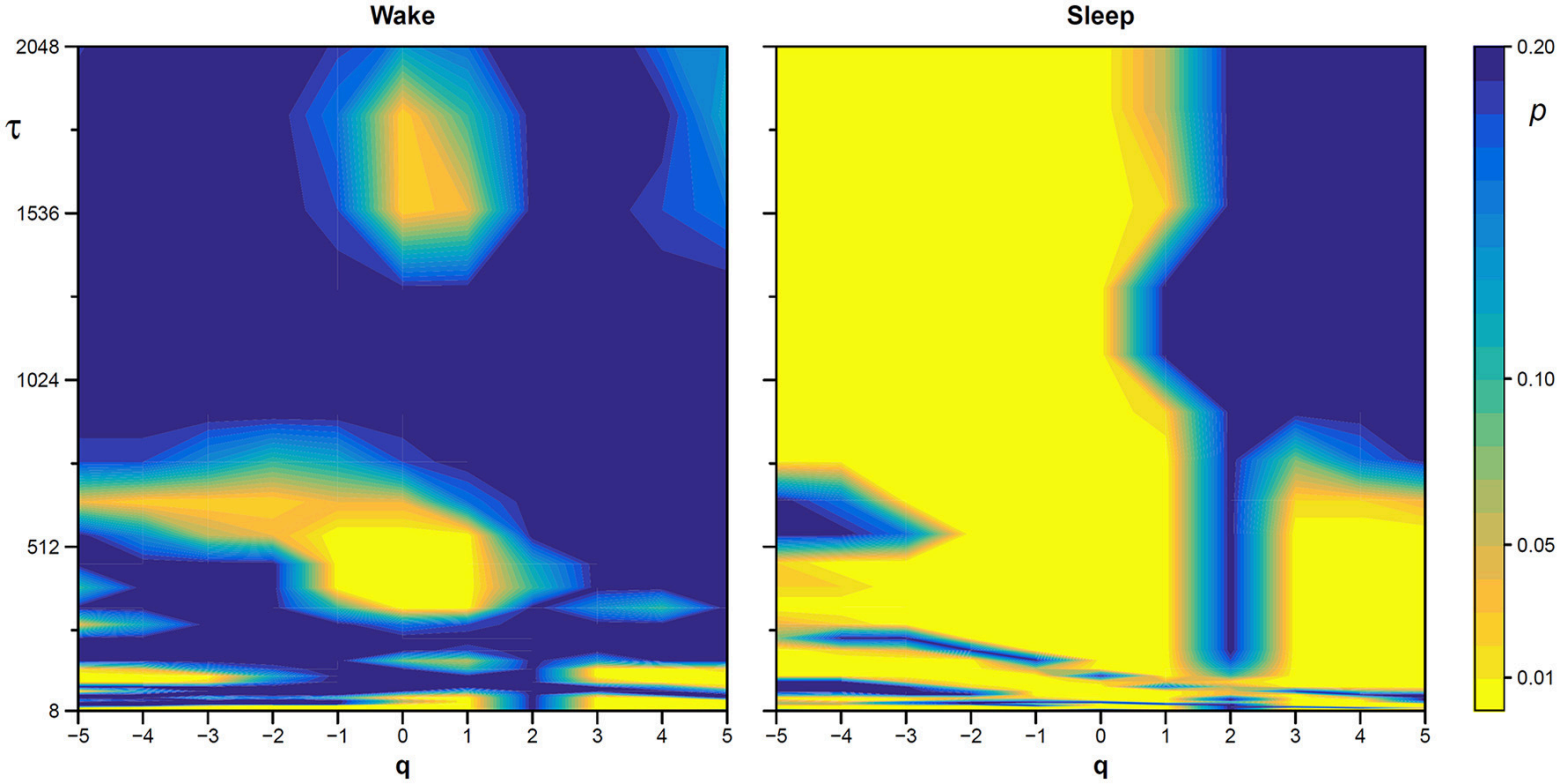
Statistical significance of the nonlinearity test on scale coefficients of heart rate. Each point represents the two-sided, type-I error probability to reject the null hypothesis comparing scale coefficients of the original series and of Fourier-phase shuffled surrogate series at each scale τ and at each moment-order *q*; differences significant at *p* < 0.01 are indicated in yellow. The temporal scale τ, in seconds, is obtained multiplying the box size, *n*, by the mean R-R interval (see Figure 8).

The statistical significance of the difference between the original and surrogate series was assessed evaluating in which percentile of the surrogate dataset the α(*q*,τ) coefficients of the original series fall. Figure 10 plots the significance *p* at each scale and moment order. The figure demonstrates the presence of a multifractal dynamics that does not depend on the linear characteristics of the power spectrum, and which characterizes Sleep more than Wake. The whole surrogate analysis of the 200 phase-shuffled series took less than 10 min with our algorithm, while the traditional algorithm would take more than 18 h according to Figure 4.

### Electroencephalogram

**Figure 11:**
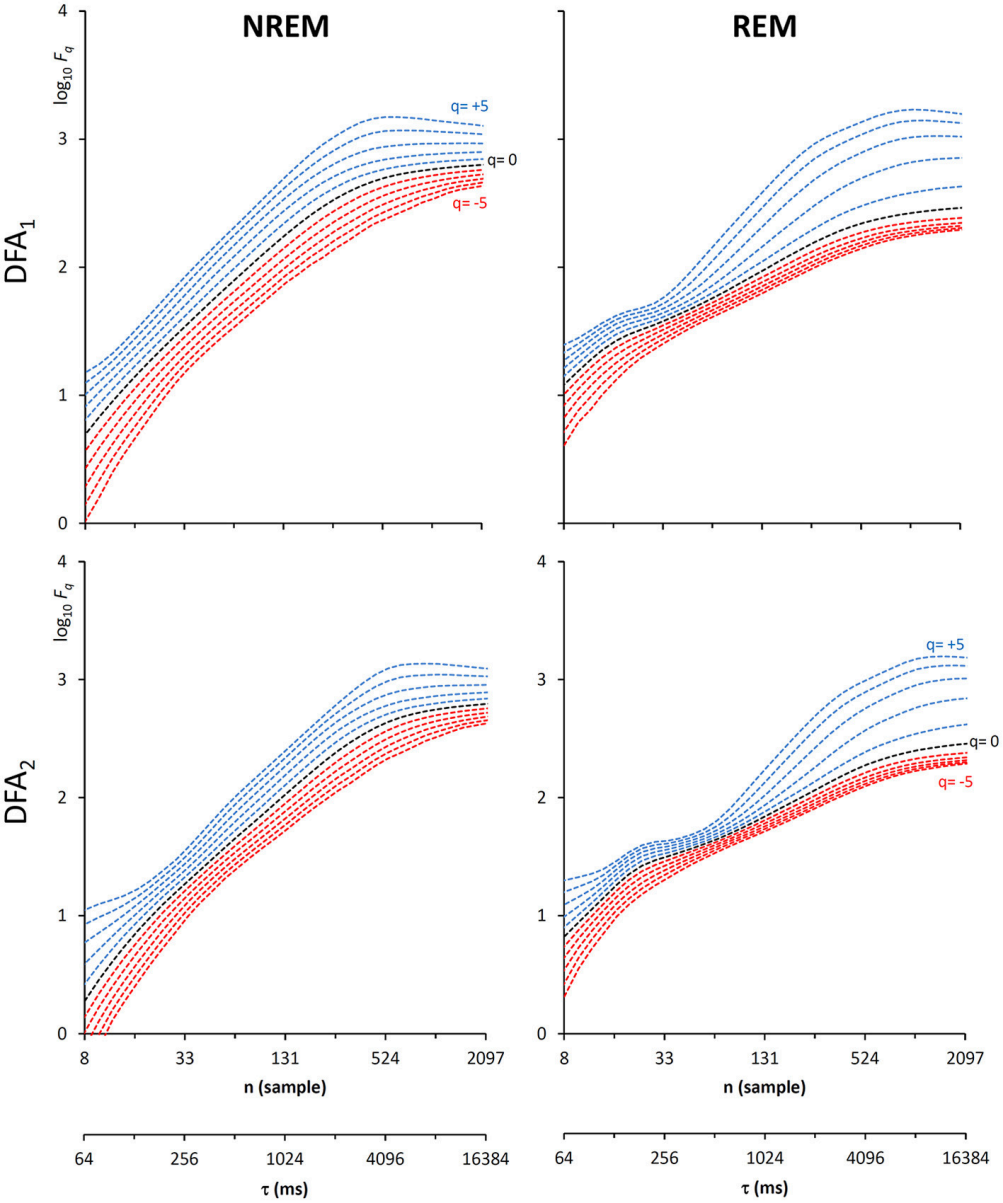
Fluctuation functions of EEG during NREM and REM sleep with maximally overlapped blocks. Time series are 1600-s segments of an EEG C4 lead sampled at 128 Hz during sleep in a healthy volunteer. The first horizontal axis is the box size, *n*, in number of samples, the second horizontal axis is the temporal scale, τ, in milliseconds, obtained multiplying *n* by the sampling period. Thresholds *Th*_1_ and *Th*_2_ as in Equations (19) and *EPS* = 10^−4^.

**Figure 12:**
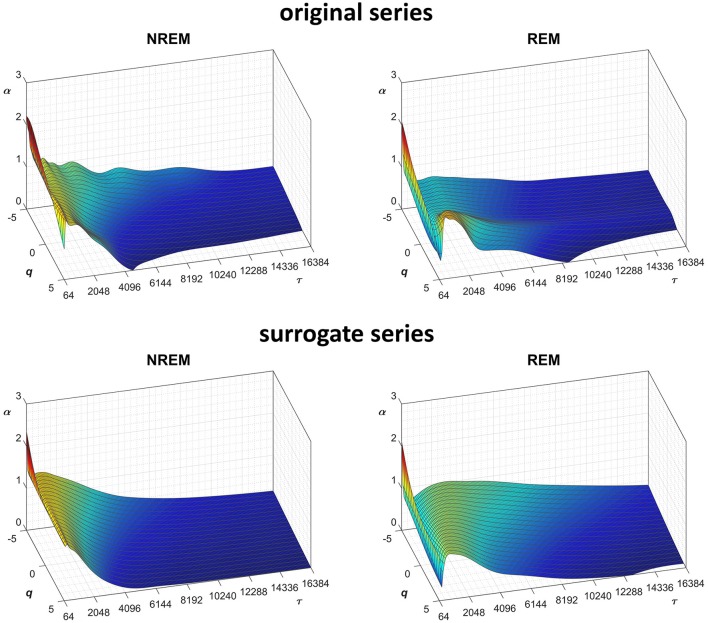
Scale coefficients during NREM and REM sleep. Coefficients calculated by Equations (22), combining DFA_1_ and DFA_2_ estimates by Equations (23), for original **(Left)** and Fourier-phase shuffled surrogate series **(Right)**. The scale τ, in milliseconds, is the box size *n* multiplied by the sampling period.

Another important field of DFA applications in physiology regards the study of EEG recordings (Hardstone et al., 2012). Traditionally, EEG is described by two (Ferree and Hwa, 2003; Abasolo et al., 2008) or three DFA coefficients (Jospin et al., 2007), but α*(n)* represented as a continuous function of the scale *n* suggests more complex structures (Castiglioni, 2011). Therefore, we considered an overnight EEG recording (C4 lead) during sleep in a healthy adult male as an additional example. The EEG signal was band-pass filtered (cut-off frequencies between 0.3 and 35 Hz) before sampling at 128 Hz, and two segments of 1,600 s duration (*N* = 204,800 samples) were selected, one during NREM and one during REM sleep. Due to the 128 Hz sampling rate, the scale *n*, expressed in number of samples, corresponds to the temporal scale τ = *n*×(1000/128) milliseconds. The fluctuation functions (Figure 11) have an almost flat profile at scales larger than 4 s, likely an effect of the filter. Furthermore, they show remarkable differences between sleep phases at shorter scales, running in parallel during NREM sleep and following a sigmoidal pattern in REM sleep. Since DFA_1_ and DFA_2_ estimates are similar, excluding the shorter scales where DFA_1_ provides a more linear increase of log *F*_*q*_*(*τ*)* with log τ, we estimated the scale coefficients with the weighted approach of Equations (23). The α(*q*,τ) surfaces (Figure 12) show a more evident multifractal structure in REM than in NREM sleep.

**Figure 13:**
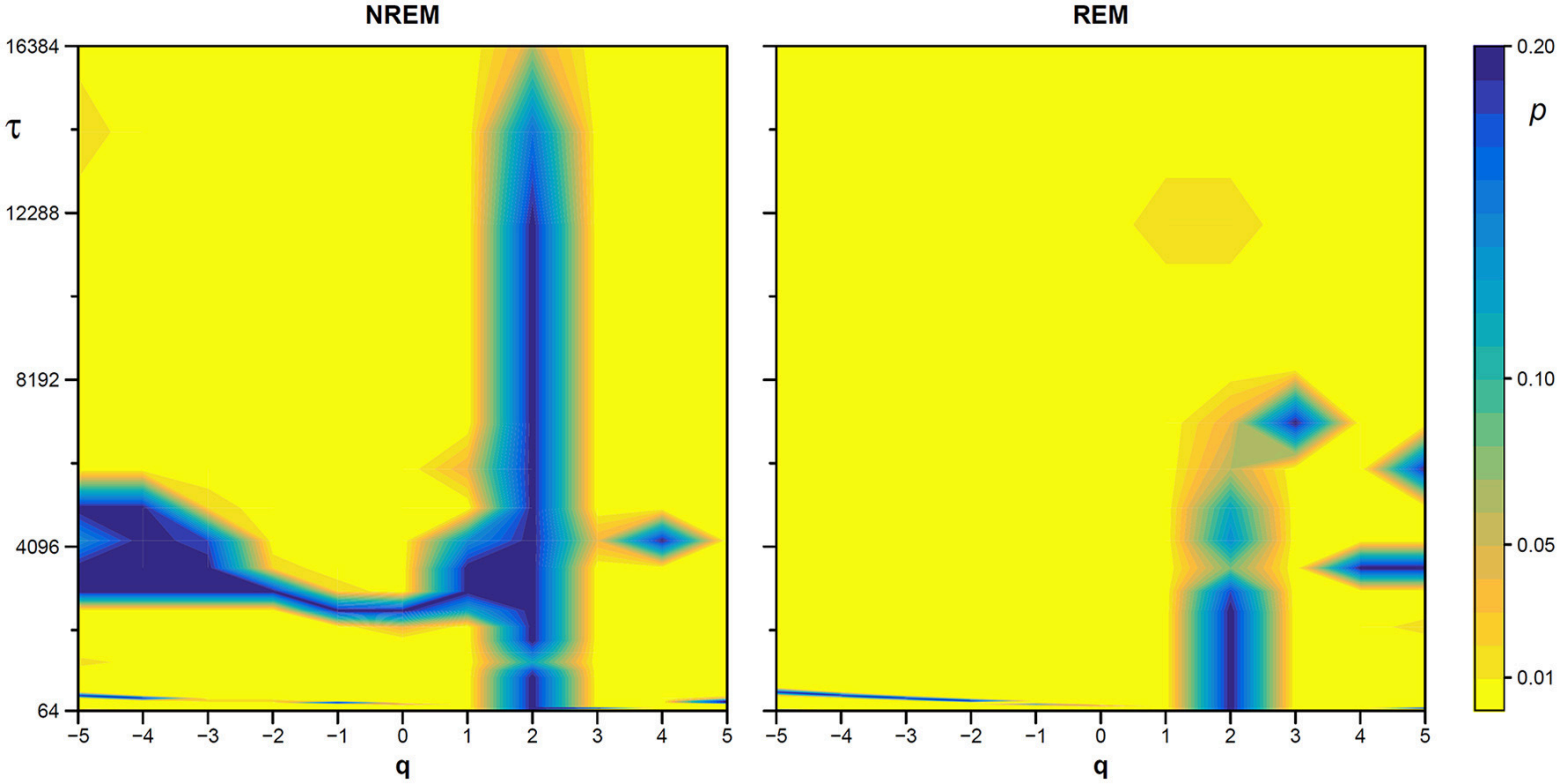
Statistical significance of nonlinearity test on EEG scale coefficients. Each point represents the two-sided, type-I error probability to reject the null hypothesis comparing scale coefficients of the original series and of Fourier-phase shuffled surrogate series at each scale τ and moment-order *q*; differences significant at *p* < 0.01 are indicated in yellow. The scale, τ, in milliseconds, is the box size *n* multiplied by the sampling period.

We also generated a surrogate dataset of 100 Fourier-phase shuffled time series for each recording, one during NREM and one during REM sleep. The average α(*q*,τ) surface of each surrogate dataset is plotted in Figure 12 for comparison with the original series. Figure 13, shows the significance of the difference between original and phase-shuffled series and indicates that the null hypothesis is rejected and nonlinearity detected almost at all scales and moment orders. The whole analysis on 200 surrogate series took 3 h and 22 min: this calculation time should be compared with the calculation time of 27 days and 8 h that the traditional algorithm would take, as from Figure 4.

## Summary of Advantages and Limitations, and Conclusions

Since the DFA introduction, hundredths of works in physiological or clinical settings quantified the fractal dynamics by α. Almost all of them provided a monofractal description, most considering only one or two ranges of scales (Perkiomaki, 2011; Sassi et al., 2015). Even if the results were promising, particularly in the field of heart rate variability, it is questioned whether a monofractal DFA estimating one or two coefficients may improve health assessment and risk stratification compared to nonfractal methods (Sassi et al., 2015). Therefore, the more advanced research in this field is investigating how DFA can be enhanced by jointly depicting the multifractal and multiscale nature of the time series in order to model pathophysiological mechanisms more completely (Zebrowski et al., 2015; Solinski et al., 2016).

In this regard, the proposed algorithm is aimed at improving scale resolution and at reducing estimation variability of the multifractal fluctuations function without the cost of an increase in calculation time that may make unfeasible the analysis of relatively long series. Therefore, we redesigned the multifractal DFA algorithm to provide estimates in a relatively short time even by using maximally overlapped blocks, with first and second-order detrending polynomials simultaneously. This allows comparing DFA_1_ and DFA_2_ estimates easily, which may be useful for evaluating the consistency of the estimates and for selecting the best detrending order. Simultaneously obtaining the DFA_1_ and DFA_2_ scale coefficients, which have a certain degree of statistical independence, also suggests new ways to improve the estimate, as in the weighted approach we proposed. The examples on real biomedical series point out that the algorithm is sufficiently fast to allow using challenging bootstrapping procedures on surrogate data that may separate linear and nonlinear components of the multiscale/multifractal dynamics. The possibility to apply such tests in clinical studies may make it easier deriving richer information to improve diagnosis or risk stratification from DFA.

Some limitations, however, should be considered. The algorithm employs detrending polynomials of first and second order only. Although these orders are used in most (if not all) DFA applications on real biosignal, the algorithm cannot be considered as general as other DFA codes. If a higher order “o” is required, the analytic expression of the polynomial regression should be derived as done in Equations (7–10) for DFA_1_ and DFA_2_, powers summations should be calculated by extending the array definition of Equation (14) and, to maintain calculation errors negligible also for DFA_o_, a new threshold *Th*_*o*_ should be defined in Equations (19), which will decrease the speed of the algorithm.

## Author Contributions

PC conceived the study and wrote the manuscript. AF wrote the code and created the figures. PC and AF developed the theoretical formulas on which the algorithm is based, designed the algorithm validation with synthesized series, made the acquisition and the analysis of real time series, interpreted the results and critically revised the manuscript.

### Conflict of Interest Statement

The authors declare that the research was conducted in the absence of any commercial or financial relationships that could be construed as a potential conflict of interest.

## Appendix

Given the time series *y*_i_ with index *i* from 1 to *N*, let's shift *i* by the integer quantity *J* so that each sample *y*_i_ is associated with the new index *j* = *i-J* running from 1-*J* to *N-J*.

The average i = $\frac{1}{n}\overset{J+n}{\underset{i=J+1}{\sum }}$ i can be rewritten as

(A1) $$\begin{array}{r} \bar{i}=\frac{1}{n}\overset{n}{\underset{j=1}{\sum }}\left(j+J\right)=\\ =\frac{1}{n}\overset{n}{\underset{j=1}{\sum }}j+J=\\ =\bar{j}+J \end{array}$$

Similarly, the summation *s*_*iy*_ = $\frac{1}{n}\overset{J+n}{\underset{i=J+1}{\sum }}iy_{i}$ in Equation (8c) can be rewritten as

(A2) $$\begin{array}{r} s_{iy}=\frac{1}{n}\overset{n}{\underset{j=1}{\sum }}\left(j+J\right)y_{i}=\\ =\frac{1}{n}\overset{n}{\underset{j=1}{\sum }}jy_{i}+J\times \bar{y}=\\ =s_{jy}+J\times \bar{y} \end{array}$$

and $s_{i^{2}y}$ = $\frac{1}{n}\overset{J+n}{\underset{i=J+1}{\sum }}i^{2}y_{i}$ in Equation (10d) as

(A3) $$\begin{array}{r} s_{i^{2}y}=\frac{1}{n}\overset{n}{\underset{j=1}{\sum }}{\left(j+J\right)}^{2}y_{i}=\\ =\frac{1}{n}\overset{n}{\underset{j=1}{\sum }}j^{2}y_{i}+2J\frac{1}{n}\overset{n}{\underset{j=1}{\sum }}jy_{i}+J^{2}\frac{1}{n}\overset{n}{\underset{j=1}{\sum }}jy_{i}\\ =s_{j^{2}y}+\left(2J+J^{2}\right)\times s_{jy} \end{array}$$

Through Equations (A1–3), *s*_*jy*_ and $s_{j^{2}y}$ can be obtained from *s*_*iy*_ and $s_{i^{2}y}$ as calculated in Equation (15).
