# Supplementary material for: A Fast DFA Algorithm for Multifractal Multiscale Analysis of Physiological Time Series
Source: Front Physiol. 2019 Mar 1;10:115. doi: 10.3389/fphys.2019.00115 (PMC6405643; doi:10.3389/fphys.2019.00115)
Supplement: Supplementary file 1 [file Data_Sheet_1.ZIP › FMFDFA.html]

FMFDFA 

## Contents

- Input parameters
- Other parameters
- Preliminary operations
- Fq calculation
- Variance of residuals computation
- Variance of residuals for linear regression
- Variance of residuals for quadratic regression
- DFA calculation for linear regression
- DFA calculation for quadratic regression
- Local Functions

```
function [bs, Fq1, Fq2] = FMFDFA(signal, q, MinBox, BoxSizeDensity, sliding, LogScaleBoxSize)
```

```
%FMFDFA Fast Multifractal DFA of first- and second-order.
%
% Copyright (C) 2019 Andrea Faini and Paolo Castiglioni
%
% This program is free software: you can redistribute it and/or modify
% it under the terms of the GNU General Public License as published by
% the Free Software Foundation, either version 3 of the License, or
% (at your option) any later version.
%
% This program is distributed in the hope that it will be useful,
% but WITHOUT ANY WARRANTY; without even the implied warranty of
% MERCHANTABILITY or FITNESS FOR A PARTICULAR PURPOSE.  See the
% GNU General Public License for more details.
%
% You should have received a copy of the GNU General Public License
% along with this program.  If not, see <http://www.gnu.org/licenses/>.
%
% Authors: Andrea Faini and Paolo Castiglioni
%
% This method is described in:
% Castiglioni P., Faini A. (2019). "A Fast DFA algorithm for multifractal
% multiscale analysis of physiological time series", Frontiers in Physiology,
% Specialty Section: Computational Physiology and Medicine
%
% Please cite the above publication when referencing this material.
%
% [bs, Fq1] = FMFDFA(signal) returns the BoxSize in log10 scale and the
% traditional non overlapped first-order DFA in log10 scale for a time
% series of N samples.
%
% This DFA algorithm is sufficiently fast to evaluate the multifractal DFA
% with maximally overlapped blocks even on long time series, as usually
% recorded in physiological or clinical settings.
%
% [bs, Fq1] = FMFDFA(signal, q) performs the first order DFA for the scale
% indicates in q (e.g. 0, -5:1:5, [-5, 2, 5]). If q is empty, it defaults
% to 2.
%
% [bs, Fq1] = FMFDFA(signal, q, MinBox) the first data block of box size
% starts from MinBox. If MinBox is empty, it defaults to 6.
%
% [bs, Fq1] = FMFDFA(signal, q, MinBox, BoxSizeDensity) set the density of
% evenly space data block. If BoxSizeDensity is empty, it defaults to 4.
%
% By default, the Box Size calculation is performed in log2 scale, in this
% case the BoxSizeDensity points out how many blocks are present in each
% doubling of box size.
%
% [bs, Fq1] = FMFDFA(signal, q, MinBox, BoxSizeDensity, sliding) performs
% the first-order DFA calculation for 'sliding' overlapped samples between
% consecutive blocks. If sliding is 0, the calculation is performed without
% overlapping, if it is 1, the overlapping is maximum. See 'L' parameter in
% eq.3 in "Fast Fq(n) calculation" section. If sliding is empty, it
% defaults to 0.
%
% [bs, Fq1] = FMFDFA(..., LogScaleBoxSize)
% if LogScaleBoxSize is 1 the box size is in logscale, otherwise it is in
% normal scale.
%
% If BoxSizeDensity is 1 and the LogScaleBoxSize is not 1, the DFA
% evaluation is performed for all blocks (with or without overlapping,
% according to 'sliding' parameter).
%
% [bs, Fq1, Fq2] = FMFDFA(...) also provides the second-order DFA in log10
% scale
%
% Copyright (C) 2019 Andrea Faini and Paolo Castiglioni
```

## Input parameters

```
if nargin < 1 || isempty(signal)
    error('Need nonempty signal')
end

if nargin < 2 || isempty(q)
    q = 2;
    warning('q set to 2');
end

if nargin < 3 || isempty(MinBox)
    MinBox = 6;
    warning('MinBox set to 6');
end

if nargin < 4 || isempty(BoxSizeDensity)
    BoxSizeDensity = 4;
    warning('BoxSizeDensity set to 4');
elseif  BoxSizeDensity < 1
    BoxSizeDensity = 4;
    warning('BoxSizeDensity set to 4');
end

if nargin < 5 || isempty(sliding)
    sliding = 0;
    warning('sliding set to 0: no overlapping');
elseif  sliding < 0
    sliding = 0;
    warning('sliding set to 0: no overlapping');
end

if nargin < 6 || isempty(LogScaleBoxSize)
    LogScaleBoxSize = 1;
    warning('LogScaleBoxSize set to 1');
end
```

## Other parameters

```
% Length of block to reduce the round-off error (details are reported in
% "Calculation precision" section)
block = 128;

% DFA1 threshold (eq.19a)
th1fx = @(n) max(n/10^8, 10^-3);

% DFA2 threshold (eq.19b)
th2fx = @(n) max(n/10^4, 10^-2);

% EPS discard variance of residuals lower than an EPS (details are reported in
% "Calculation precision" section)
eps = 10^-4;

% Perform DFA2 ?
if nargout > 2
    quadRreg = 1; % yes
else
    quadRreg = 0; % no
end
```

## Preliminary operations

```
% Anonymous functions for eqs.12
sumPow1 = @(n) n*(n+1)/2;
sumPow2 = @(n) n^3/3 + n^2/2 + n/6;
sumPow3 = @(n) n^4/4 + n^3/2 + n^2/4;
sumPow4 = @(n) n^5/5 + n^4/2 + n^3/3 - n/30;

% Anonymous functions for eqs.8b,d
fi = @(z) z; % Identify function
fx2y = @(z) z(1,:).^2 .* z(2,:); % x^2y function

% Column Major
if size(signal,2) == 1
    signal = transpose(signal);
end

% This scale factor is used to evenly space the ouput on a log scale
LogScaleFactor = 2 ^ (1 / BoxSizeDensity);

% Length of signal
N = length(signal);
x = 1:N;

% Cumulative sum of normalized series (eq.1-2)
y = cumsum(zscore(signal));
% Standard deviation of orignal series to adjust the Fq (details are
% reported in "Fast Fq(n) calculation" section)
sh = log10(std(signal));

% Matrices of sum of products (see eqs.15,17-18)
vSy = CumSumBlock(block,fi,y); % eq.8b
vSxy = CumSumBlock(block,@prod,x,y); % eq.8c
vSy2 = CumSumBlock(block,@prod,y,y); % term in eqs.16a-b
% Only for quadratic regression
if quadRreg == 1
    vSx2y = CumSumBlock(block,fx2y,x,y); % eq.8d
end

% Set the biggest window to 1/4 the data length, this can be adjusted slightly
MaxBox = fix(0.25 * N);

% Cumputation of threshold 1 and 2
th1 = th1fx(N);
th2 = th2fx(N);
```

## Fq calculation

Now find the best fit lines and find fluctuation about the line loop for each box size from MinBox to MaxBox

```
TempBoxSize = MinBox;
BoxSize = MinBox;

bs = [];
Fq1 = [];
Fq2 = [];

while BoxSize <= MaxBox
```

```
    % Overlapping ?
    if sliding == 0
        Shift = BoxSize; % No overlapping
    else
        Shift = sliding; % Overlapping with shift interval
    end

    % M blocks
    M = fix(1 + (N - BoxSize) / Shift); % eq.3

    % Preallocation space for the variance of residuals array
    Sigma2vreg1 = zeros(1, M); % Linear regression
    % Only for quadratic regression
    if quadRreg == 1
        Sigma2vreg2 = zeros(1, M); % Quadratic regression
    end

    % Indices for variance of residuals array
    nS1=1;
    nS2=1;

    % Sums of power of BoxSize consecutive integer
    SX = sumPow1(BoxSize); % eq.12a
    SX2 = sumPow2(BoxSize); % eq.12b
    % Only for quadratic regression
    if quadRreg == 1
        SX3 = sumPow3(BoxSize); % eq.12c
        SX4 = sumPow4(BoxSize); % eq.12d
    end
```

## Variance of residuals computation

```
    for ii = 1:M
```

```
        % Find the indices (P1 and P2) for the eq.18
        P1 = 1 + (ii - 1) * Shift;
        P2 = P1 + BoxSize;

        p1 = (mod(P1,block)>=0)*(P1-1) + 1;
        a = ceil(P1/block);
        b = floor((P2-1)/block);
        if a<=b
            ps = a * block : block : b * block;
        else
            ps = 1;
        end
```

## Variance of residuals for linear regression

```
        SY = -vSy(p1) + vSy(P2) + sum(vSy(ps)); % eq.8b
        SXY = -vSxy(p1) + vSxy(P2) + sum(vSxy(ps)) - (P1-1) * SY; % eq.8c
        SY2 = -vSy2(p1) + vSy2(P2) + sum(vSy2(ps));% term in eqs.16a-b

        % Coefficients of eq.6a
        Areg1 = (BoxSize * SXY - SX * SY) / (BoxSize * SX2 - SX ^ 2);
        Breg1 = (SY - Areg1 * SX) / BoxSize;

        % Variance of residuals for the current box (eq.16a)
        Sigma2reg1 = SY2 - 2 * Areg1 * SXY - 2 * Breg1 * SY + (Areg1 ^ 2) * SX2 + 2 * Areg1 * Breg1 * SX + (Breg1 ^ 2) * BoxSize;

        % If the variance of residuals is lower than the threshold
        % it is recalculated directly (details are reported in "Calculation
        % precision" section)
        if Sigma2reg1 <= th1

            [Areg1,  Breg1] = CoeffReg(1:BoxSize,y(P1:P2-1),1);

            Sigma2reg1 = 0;
            for iii = 1 : BoxSize
                Sigma2reg1 = Sigma2reg1 + (y(P1 + iii -1) - Areg1 * iii - Breg1)^2; % eq.16a
            end
        end

        % If the variance of residuals is lower than EPS threshold
        % it is discared (details are reported in "Calculation
        % precision" section)
        if Sigma2reg1 > eps
            Sigma2reg1 = Sigma2reg1 / BoxSize;
            Sigma2vreg1(nS1) = Sigma2reg1;
            nS1=nS1+1;
        end
```

## Variance of residuals for quadratic regression

```
        if quadRreg == 1

            % eq.10d
            SX2Y = (-vSx2y(p1) + vSx2y(P2) + sum(vSx2y(ps))) - 2 * (P1 - 1) * (-vSxy(p1) + vSxy(P2) + sum(vSxy(ps))) + (P1-1)^2 * SY;

            % Denominator for eqs.9a-b
            D = ((BoxSize * SX2 - SX ^ 2) * (BoxSize * SX4 - SX2 ^ 2) - (BoxSize * SX3 - SX * SX2) ^ 2);


            % Coefficients of eq.6b
            if  D > 0

                Breg2 = ((BoxSize * SXY - SX * SY) * (BoxSize * SX4 - SX2 ^2) - (BoxSize * SX2Y - SX2 * SY) * (BoxSize * SX3 - SX * SX2)) / ...
                    D;
                Areg2 = ((BoxSize * SX2Y - SX2 * SY) * (BoxSize * SX2 - SX ^2) - (BoxSize * SXY - SX * SY) * (BoxSize * SX3 - SX * SX2)) / ...
                    D;
                Creg2 = (SY - Breg2 * SX - Areg2 * SX2) / BoxSize;

                % Variance of residuals for the current box (eq.16b)
                Sigma2reg2 = SY2 + (Areg2 ^ 2) * SX4 + (Breg2 ^ 2) * SX2 + (Creg2 ^ 2) * BoxSize ...
                    - 2 * Areg2 * SX2Y - 2 * Breg2 * SXY - 2 * Creg2 * SY ...
                    + 2 * Areg2 * Breg2 * SX3 + 2 * Areg2 * Creg2 * SX2 + 2 * Breg2 *  Creg2 * SX;
            else
                Sigma2reg2=0;
            end

            % If the variance of residuals is lower than the threshold
            % it is recalculated directly (details are reported in "Calculation
            % precision" section)
            if Sigma2reg2 <= th2

                [Areg2, Breg2, Creg2] = CoeffReg(1:BoxSize,y(P1:P2-1),2);

                Sigma2reg2 = 0;
                for iii = 1 : BoxSize
                    Sigma2reg2 = Sigma2reg2 + (y(P1 + iii - 1) - Areg2 * iii.^2 - Breg2 * iii - Creg2)^2; % eq.16b
                end
            end

            % If the variance of residuals is lower than EPS threshold
            % it is discared (details are reported in "Calculation
            % precision" section
            if Sigma2reg2 > eps
                Sigma2reg2 = Sigma2reg2 / BoxSize;
                Sigma2vreg2(nS2) = Sigma2reg2;
                nS2=nS2+1;
            end
        end
```

```
    end
```

## DFA calculation for linear regression

```
    Fluctuation1 = zeros(1,length(q));

    for iq = 1:length(q)

        Q = q(iq);
        Sigma2vreg1(nS1:end) = [];
        L = length(Sigma2vreg1);
        if Q == 0
            Fluctuation1(iq) = sum(log(Sigma2vreg1));
            Fluctuation1(iq) = exp(Fluctuation1(iq) / (2 * L));
        else
            Fluctuation1(iq) = sum(Sigma2vreg1 .^ (Q / 2));
            Fluctuation1(iq) = (Fluctuation1(iq) / L) ^ (1 / Q);
        end

    end

    % Output for DFA1
    bs = [bs, log10(BoxSize)];
    Fq1 = [Fq1, log10(Fluctuation1') + sh]; % sh, see details are reported
    % in "Fast Fq(n) calculation" section
```

## DFA calculation for quadratic regression

```
    if quadRreg == 1

        Fluctuation2 = zeros(1,length(q));

        for iq = 1:length(q)

            Q = q(iq);

           Sigma2vreg2(nS2:end) = [];
            L = length(Sigma2vreg2);
            if Q == 0
                Fluctuation2(iq) = sum(log(Sigma2vreg2));
                Fluctuation2(iq) = exp(Fluctuation2(iq) / (2 * L));
            else
                Fluctuation2(iq) = sum(Sigma2vreg2 .^ (Q / 2));
                Fluctuation2(iq) = (Fluctuation2(iq) / L) ^ (1 / Q);
            end

        end

        % Output for DF2
        Fq2 = [Fq2, log10(Fluctuation2') + sh]; % sh, details are reported
        % in "Fast Fq(n) calculation" section
    end

    % Update the box size
    if LogScaleBoxSize==1
        TempBoxSize = TempBoxSize * LogScaleFactor;
        while TempBoxSize < (BoxSize + 1)
            TempBoxSize = TempBoxSize * LogScaleFactor;
        end
        BoxSize = round(TempBoxSize);
    else
        BoxSize = BoxSize + BoxSizeDensity;
    end
```

```
end
```

```
end
```

## Local Functions

```
function [A, B, C] = CoeffReg(x,y,ord)

if  ord < 0 || ord > 2
    error('Set ord 1 or 2');
end

N = length(x);
mx = mean(x);
my = mean(y);

Sxx = 0;
for i = 1:N
    Sxx = Sxx + (x(i) - mx)^2;
end
Sxx = Sxx/N;

Sxy = 0;
for i = 1:N
    Sxy = Sxy + (x(i) - mx) * (y(i) - my);
end
Sxy = Sxy/N;

% Linear regression
if ord == 1
    A = Sxy/Sxx;
    B = my - A * mx;
    C = NaN;
    return
end

% Quadratic regression
mx2 = mean(x.^2);

Sxx2 = 0;
for i = 1:N
    Sxx2 = Sxx2 + (x(i) - mx2) * (x(i)^2 - mx2);
end
Sxx2 = Sxx2/N;

Sx2x2 = 0;
for i = 1:N
    Sx2x2 = Sx2x2 + (x(i)^2 - mx2)^2;
end
Sx2x2 = Sx2x2/N;

Sx2y = 0;
for i = 1:N
    Sx2y = Sx2y + (x(i)^2 - mx2) * (y(i) - my); % o alternativa?
end
Sx2y = Sx2y/N;

Den = Sxx * Sx2x2 - (Sxx2)^2;

B = (Sxy * Sx2x2 - Sx2y * Sxx2)/Den;

A = (Sx2y * Sxx - Sxy * Sxx2)/Den;

C = my - B * mx - A * mx2;
end

function M = CumSumBlock(block, fx, varargin)

mat = zeros(length(varargin),numel(varargin{1})+1);

% The first point is 0
mat(1,:) = [0, varargin{1}];

for v = 2:length(varargin)
    mat(v,:) = [0, varargin{v}];
end

N = size(mat,2);

% Number fo blocks
m = ceil(N/block);

% Preallocation space matrix n x m
M = zeros(N,1);

% Fill the matrix
for i = 1:m
    if (i*block)>N
        d = i*block - N;
    else
        d = 0;
    end
    l = (i-1)*block+1:(i*block) - d;
    M(l) = cumsum(fx(mat(:,l)));
end
end
```

Published with MATLAB® R2017b
