# Supplementary material for: A Fast DFA Algorithm for Multifractal Multiscale Analysis of Physiological Time Series
Source: Front Physiol. 2019 Mar 1;10:115. doi: 10.3389/fphys.2019.00115 (PMC6405643; doi:10.3389/fphys.2019.00115)
Supplement: Supplementary file 1 [file Data_Sheet_1.ZIP › GNU General Public License.rtf]

GNU Free Documentation License
Version 1.3, 3 November 2008
Copyright © 2000, 2001, 2002, 2007, 2008 Free Software Foundation, Inc. <https://fsf.org/>
Everyone is permitted to copy and distribute verbatim copies of this license document, but changing it is not allowed.
0. PREAMBLE
The purpose of this License is to make a manual, textbook, or other functional and useful document "free" in the sense of freedom: to assure everyone the effective freedom to copy and redistribute it, with or without modifying it, either commercially or noncommercially. Secondarily, this License preserves for the author and publisher a way to get credit for their work, while not being considered responsible for modifications made by others.
This License is a kind of "copyleft", which means that derivative works of the document must themselves be free in the same sense. It complements the GNU General Public License, which is a copyleft license designed for free software.
We have designed this License in order to use it for manuals for free software, because free software needs free documentation: a free program should come with manuals providing the same freedoms that the software does. But this License is not limited to software manuals; it can be used for any textual work, regardless of subject matter or whether it is published as a printed book. We recommend this License principally for works whose purpose is instruction or reference.
1. APPLICABILITY AND DEFINITIONS
This License applies to any manual or other work, in any medium, that contains a notice placed by the copyright holder saying it can be distributed under the terms of this License. Such a notice grants a world-wide, royalty-free license, unlimited in duration, to use that work under the conditions stated herein. The "Document", below, refers to any such manual or work. Any member of the public is a licensee, and is addressed as "you". You accept the license if you copy, modify or distribute the work in a way requiring permission under copyright law.
A "Modified Version" of the Document means any work containing the Document or a portion of it, either copied verbatim, or with modifications and/or translated into another language.
A "Secondary Section" is a named appendix or a front-matter section of the Document that deals exclusively with the relationship of the publishers or authors of the Document to the Document's overall subject (or to related matters) and contains nothing that could fall directly within that overall subject. (Thus, if the Document is in part a textbook of mathematics, a Secondary Section may not explain any mathematics.) The relationship could be a matter of historical connection with the subject or with related matters, or of legal, commercial, philosophical, ethical or political position regarding them.
The "Invariant Sections" are certain Secondary Sections whose titles are designated, as being those of Invariant Sections, in the notice that says that the Document is released under this License. If a section does not fit the above definition of Secondary then it is not allowed to be designated as Invariant. The Document may contain zero Invariant Sections. If the Document does not identify any Invariant Sections then there are none.
The "Cover Texts" are certain short passages of text that are listed, as Front-Cover Texts or Back-Cover Texts, in the notice that says that the Document is released under this License. A Front-Cover Text may be at most 5 words, and a Back-Cover Text may be at most 25 words.
A "Transparent" copy of the Document means a machine-readable copy, represented in a format whose specification is available to the general public, that is suitable for revising the document straightforwardly with generic text editors or (for images composed of pixels) generic paint programs or (for drawings) some widely available drawing editor, and that is suitable for input to text formatters or for automatic translation to a variety of formats suitable for input to text formatters. A copy made in an otherwise Transparent file format whose markup, or absence of markup, has been arranged to thwart or discourage subsequent modification by readers is not Transparent. An image format is not Transparent if used for any substantial amount of text. A copy that is not "Transparent" is called "Opaque".
Examples of suitable formats for Transparent copies include plain ASCII without markup, Texinfo input format, LaTeX input format, SGML or XML using a publicly available DTD, and standard-conforming simple HTML, PostScript or PDF designed for human modification. Examples of transparent image formats include PNG, XCF and JPG. Opaque formats include proprietary formats that can be read and edited only by proprietary word processors, SGML or XML for which the DTD and/or processing tools are not generally available, and the machine-generated HTML, PostScript or PDF produced by some word processors for output purposes only.
The "Title Page" means, for a printed book, the title page itself, plus such following pages as are needed to hold, legibly, the material this License requires to appear in the title page. For works in formats which do not have any title page as such, "Title Page" means the text near the most prominent appearance of the work's title, preceding the beginning of the body of the text.
The "publisher" means any person or entity that distributes copies of the Document to the public.
A section "Entitled XYZ" means a named subunit of the Document whose title either is precisely XYZ or contains XYZ in parentheses following text that translates XYZ in another language. (Here XYZ stands for a specific section name mentioned below, such as "Acknowledgements", "Dedications", "Endorsements", or "History".) To "Preserve the Title" of such a section when you modify the Document means that it remains a section "Entitled XYZ" according to this definition.
The Document may include Warranty Disclaimers next to the notice which states that this License applies to the Document. These Warranty Disclaimers are considered to be included by reference in this License, but only as regards disclaiming warranties: any other implication that these Warranty Disclaimers may have is void and has no effect on the meaning of this License.
2. VERBATIM COPYING
You may copy and distribute the Document in any medium, either commercially or noncommercially, provided that this License, the copyright notices, and the license notice saying this License applies to the Document are reproduced in all copies, and that you add no other conditions whatsoever to those of this License. You may not use technical measures to obstruct or control the reading or further copying of the copies you make or distribute. However, you may accept compensation in exchange for copies. If you distribute a large enough number of copies you must also follow the conditions in section 3.
You may also lend copies, under the same conditions stated above, and you may publicly display copies.
3. COPYING IN QUANTITY
If you publish printed copies (or copies in media that commonly have printed covers) of the Document, numbering more than 100, and the Document's license notice requires Cover Texts, you must enclose the copies in covers that carry, clearly and legibly, all these Cover Texts: Front-Cover Texts on the front cover, and Back-Cover Texts on the back cover. Both covers must also clearly and legibly identify you as the publisher of these copies. The front cover must present the full title with all words of the title equally prominent and visible. You may add other material on the covers in addition. Copying with changes limited to the covers, as long as they preserve the title of the Document and satisfy these conditions, can be treated as verbatim copying in other respects.
If the required texts for either cover are too voluminous to fit legibly, you should put the first ones listed (as many as fit reasonably) on the actual cover, and continue the rest onto adjacent pages.
If you publish or distribute Opaque copies of the Document numbering more than 100, you must either include a machine-readable Transparent copy along with each Opaque copy, or state in or with each Opaque copy a computer-network location from which the general network-using public has access to download using public-standard network protocols a complete Transparent copy of the Document, free of added material. If you use the latter option, you must take reasonably prudent steps, when you begin distribution of Opaque copies in quantity, to ensure that this Transparent copy will remain thus accessible at the stated location until at least one year after the last time you distribute an Opaque copy (directly or through your agents or retailers) of that edition to the public.
It is requested, but not required, that you contact the authors of the Document well before redistributing any large number of copies, to give them a chance to provide you with an updated version of the Document.
4. MODIFICATIONS
You may copy and distribute a Modified Version of the Document under the conditions of sections 2 and 3 above, provided that you release the Modified Version under precisely this License, with the Modified Version filling the role of the Document, thus licensing distribution and modification of the Modified Version to whoever possesses a copy of it. In addition, you must do these things in the Modified Version:
•	A. Use in the Title Page (and on the covers, if any) a title distinct from that of the Document, and from those of previous versions (which should, if there were any, be listed in the History section of the Document). You may use the same title as a previous version if the original publisher of that version gives permission.
•	B. List on the Title Page, as authors, one or more persons or entities responsible for authorship of the modifications in the Modified Version, together with at least five of the principal authors of the Document (all of its principal authors, if it has fewer than five), unless they release you from this requirement.
•	C. State on the Title page the name of the publisher of the Modified Version, as the publisher.
•	D. Preserve all the copyright notices of the Document.
•	E. Add an appropriate copyright notice for your modifications adjacent to the other copyright notices.
•	F. Include, immediately after the copyright notices, a license notice giving the public permission to use the Modified Version under the terms of this License, in the form shown in the Addendum below.
•	G. Preserve in that license notice the full lists of Invariant Sections and required Cover Texts given in the Document's license notice.
•	H. Include an unaltered copy of this License.
•	I. Preserve the section Entitled "History", Preserve its Title, and add to it an item stating at least the title, year, new authors, and publisher of the Modified Version as given on the Title Page. If there is no section Entitled "History" in the Document, create one stating the title, year, authors, and publisher of the Document as given on its Title Page, then add an item describing the Modified Version as stated in the previous sentence.
•	J. Preserve the network location, if any, given in the Document for public access to a Transparent copy of the Document, and likewise the network locations given in the Document for previous versions it was based on. These may be placed in the "History" section. You may omit a network location for a work that was published at least four years before the Document itself, or if the original publisher of the version it refers to gives permission.
•	K. For any section Entitled "Acknowledgements" or "Dedications", Preserve the Title of the section, and preserve in the section all the substance and tone of each of the contributor acknowledgements and/or dedications given therein.
•	L. Preserve all the Invariant Sections of the Document, unaltered in their text and in their titles. Section numbers or the equivalent are not considered part of the section titles.
•	M. Delete any section Entitled "Endorsements". Such a section may not be included in the Modified Version.
•	N. Do not retitle any existing section to be Entitled "Endorsements" or to conflict in title with any Invariant Section.
•	O. Preserve any Warranty Disclaimers.
If the Modified Version includes new front-matter sections or appendices that qualify as Secondary Sections and contain no material copied from the Document, you may at your option designate some or all of these sections as invariant. To do this, add their titles to the list of Invariant Sections in the Modified Version's license notice. These titles must be distinct from any other section titles.
You may add a section Entitled "Endorsements", provided it contains nothing but endorsements of your Modified Version by various parties—\for example, statements of peer review or that the text has been approved by an organization as the authoritative definition of a standard.
You may add a passage of up to five words as a Front-Cover Text, and a passage of up to 25 words as a Back-Cover Text, to the end of the list of Cover Texts in the Modified Version. Only one passage of Front-Cover Text and one of Back-Cover Text may be added by (or through arrangements made by) any one entity. If the Document already includes a cover text for the same cover, previously added by you or by arrangement made by the same entity you are acting on behalf of, you may not add another; but you may replace the old one, on explicit permission from the previous publisher that added the old one.
The author(s) and publisher(s) of the Document do not by this License give permission to use their names for publicity for or to assert or imply endorsement of any Modified Version.
5. COMBINING DOCUMENTS
You may combine the Document with other documents released under this License, under the terms defined in section 4 above for modified versions, provided that you include in the combination all of the Invariant Sections of all of the original documents, unmodified, and list them all as Invariant Sections of your combined work in its license notice, and that you preserve all their Warranty Disclaimers.
The combined work need only contain one copy of this License, and multiple identical Invariant Sections may be replaced with a single copy. If there are multiple Invariant Sections with the same name but different contents, make the title of each such section unique by adding at the end of it, in parentheses, the name of the original author or publisher of that section if known, or else a unique number. Make the same adjustment to the section titles in the list of Invariant Sections in the license notice of the combined work.
In the combination, you must combine any sections Entitled "History" in the various original documents, forming one section Entitled "History"; likewise combine any sections Entitled "Acknowledgements", and any sections Entitled "Dedications". You must delete all sections Entitled "Endorsements".
6. COLLECTIONS OF DOCUMENTS
You may make a collection consisting of the Document and other documents released under this License, and replace the individual copies of this License in the various documents with a single copy that is included in the collection, provided that you follow the rules of this License for verbatim copying of each of the documents in all other respects.
You may extract a single document from such a collection, and distribute it individually under this License, provided you insert a copy of this License into the extracted document, and follow this License in all other respects regarding verbatim copying of that document.
7. AGGREGATION WITH INDEPENDENT WORKS
A compilation of the Document or its derivatives with other separate and independent documents or works, in or on a volume of a storage or distribution medium, is called an "aggregate" if the copyright resulting from the compilation is not used to limit the legal rights of the compilation's users beyond what the individual works permit. When the Document is included in an aggregate, this License does not apply to the other works in the aggregate which are not themselves derivative works of the Document.
If the Cover Text requirement of section 3 is applicable to these copies of the Document, then if the Document is less than one half of the entire aggregate, the Document's Cover Texts may be placed on covers that bracket the Document within the aggregate, or the electronic equivalent of covers if the Document is in electronic form. Otherwise they must appear on printed covers that bracket the whole aggregate.
8. TRANSLATION
Translation is considered a kind of modification, so you may distribute translations of the Document under the terms of section 4. Replacing Invariant Sections with translations requires special permission from their copyright holders, but you may include translations of some or all Invariant Sections in addition to the original versions of these Invariant Sections. You may include a translation of this License, and all the license notices in the Document, and any Warranty Disclaimers, provided that you also include the original English version of this License and the original versions of those notices and disclaimers. In case of a disagreement between the translation and the original version of this License or a notice or disclaimer, the original version will prevail.
If a section in the Document is Entitled "Acknowledgements", "Dedications", or "History", the requirement (section 4) to Preserve its Title (section 1) will typically require changing the actual title.
9. TERMINATION
You may not copy, modify, sublicense, or distribute the Document except as expressly provided under this License. Any attempt otherwise to copy, modify, sublicense, or distribute it is void, and will automatically terminate your rights under this License.
However, if you cease all violation of this License, then your license from a particular copyright holder is reinstated (a) provisionally, unless and until the copyright holder explicitly and finally terminates your license, and (b) permanently, if the copyright holder fails to notify you of the violation by some reasonable means prior to 60 days after the cessation.
Moreover, your license from a particular copyright holder is reinstated permanently if the copyright holder notifies you of the violation by some reasonable means, this is the first time you have received notice of violation of this License (for any work) from that copyright holder, and you cure the violation prior to 30 days after your receipt of the notice.
Termination of your rights under this section does not terminate the licenses of parties who have received copies or rights from you under this License. If your rights have been terminated and not permanently reinstated, receipt of a copy of some or all of the same material does not give you any rights to use it.
10. FUTURE REVISIONS OF THIS LICENSE
The Free Software Foundation may publish new, revised versions of the GNU Free Documentation License from time to time. Such new versions will be similar in spirit to the present version, but may differ in detail to address new problems or concerns. See https://www.gnu.org/licenses/.
Each version of the License is given a distinguishing version number. If the Document specifies that a particular numbered version of this License "or any later version" applies to it, you have the option of following the terms and conditions either of that specified version or of any later version that has been published (not as a draft) by the Free Software Foundation. If the Document does not specify a version number of this License, you may choose any version ever published (not as a draft) by the Free Software Foundation. If the Document specifies that a proxy can decide which future versions of this License can be used, that proxy's public statement of acceptance of a version permanently authorizes you to choose that version for the Document.
11. RELICENSING
"Massive Multiauthor Collaboration Site" (or "MMC Site") means any World Wide Web server that publishes copyrightable works and also provides prominent facilities for anybody to edit those works. A public wiki that anybody can edit is an example of such a server. A "Massive Multiauthor Collaboration" (or "MMC") contained in the site means any set of copyrightable works thus published on the MMC site.
"CC-BY-SA" means the Creative Commons Attribution-Share Alike 3.0 license published by Creative Commons Corporation, a not-for-profit corporation with a principal place of business in San Francisco, California, as well as future copyleft versions of that license published by that same organization.
"Incorporate" means to publish or republish a Document, in whole or in part, as part of another Document.
An MMC is "eligible for relicensing" if it is licensed under this License, and if all works that were first published under this License somewhere other than this MMC, and subsequently incorporated in whole or in part into the MMC, (1) had no cover texts or invariant sections, and (2) were thus incorporated prior to November 1, 2008.
The operator of an MMC Site may republish an MMC contained in the site under CC-BY-SA on the same site at any time before August 1, 2009, provided the MMC is eligible for relicensing.
ADDENDUM: How to use this License for your documents
To use this License in a document you have written, include a copy of the License in the document and put the following copyright and license notices just after the title page:
Copyright (C)  YEAR  YOUR NAME.
Permission is granted to copy, distribute and/or modify this document under the terms of the GNU Free Documentation License, Version 1.3 or any later version published by the Free Software Foundation; with no Invariant Sections, no Front-Cover Texts, and no Back-Cover Texts. A copy of the license is included in the section entitled "GNU Free Documentation License".
If you have Invariant Sections, Front-Cover Texts and Back-Cover Texts, replace the "with …c Texts." line with this:
with the Invariant Sections being LIST THEIR TITLES, with the Front-Cover Texts being LIST, and with the Back-Cover Texts being LIST.
If you have Invariant Sections without Cover Texts, or some other combination of the three, merge those two alternatives to suit the situation.
If your document contains nontrivial examples of program code, we recommend releasing these examples in parallel under your choice of free software license, such as the GNU General Public License, to permit their use in free software.
