# Supplementary material for: A Fast DFA Algorithm for Multifractal Multiscale Analysis of Physiological Time Series
Source: Front Physiol. 2019 Mar 1;10:115. doi: 10.3389/fphys.2019.00115 (PMC6405643; doi:10.3389/fphys.2019.00115)
Supplement: Supplementary file 1 [file Data_Sheet_1.ZIP › slpMFMSDFA.html]

slpMFMSDFA 

## Contents

- Input parameters
- Compute alpha(q,n)
- Local Function

```
function [aq, bse, Fqe] = slpMFMSDFA(bs, Fq)
```

```
%slpMFMSDFA Local slope for Mulfifractal Multiscale DFA with derivative
% formula on 5 points.
%
% Copyright (C) 2019 Andrea Faini and Paolo Castiglioni
%
% This program is free software: you can redistribute it and/or modify
% it under the terms of the GNU General Public License as published by
% the Free Software Foundation, either version 3 of the License, or
% (at your option) any later version.
%
% This program is distributed in the hope that it will be useful,
% but WITHOUT ANY WARRANTY; without even the implied warranty of
% MERCHANTABILITY or FITNESS FOR A PARTICULAR PURPOSE.  See the
% GNU General Public License for more details.
%
% You should have received a copy of the GNU General Public License
% along with this program.  If not, see <http://www.gnu.org/licenses/>.
%
% Authors: Andrea Faini and Paolo Castiglioni
%
% This method is described in:
% Castiglioni P., Faini A. (2019). "A Fast DFA algorithm for multifractal
% multiscale analysis of physiological time series", Frontiers in Physiology,
% Specialty Section: Computational Physiology and Medicine
%
% Please cite the above publication when referencing this material.
%
% aq = MFMSDFA(bs, Fq) returns the local slope for a fluctuation function
% Fq over a box size series bs. The bs and Fq have to be in log scale.
% The local slopes are calculated as described in eqs.22.
%
% By default, the bs is equispaced and Fq is spline-interpolated over the
% equispaced bs before performing the local slopes.
%
% [aq, bse] = MFMSDFA(bs, Fq) returns also the equispaced bs. The bse scale
% is as the bs.
%
% [aq, bse, Fqe] = MFMSDFA(bs, Fq) returns also the equispaced Fq. The Fqe
% scale is as the Fq.
```

## Input parameters

```
if nargin < 1 || isempty(bs)
    error('Need nonempty bs')
end

if nargin < 2 || isempty(Fq)
    error('Need nonempty Fq')
end
```

## Compute alpha(q,n)

```
aq = nan(size(Fq));

% equipacing
bse = linspace(bs(1), bs(end), length(bs));
Fqe = spline(bs,Fq,bse);

% Local slopes, eqs.22
for f = 1 : size(Fq,1)
    aq(f,:) = f5p(bse, Fqe(f,:));
end
```

```
end
```

## Local Function

```
function y1 = f5p(t, f)
% See eqs.22

% Column Major
if size(t,2)==1;
    t=transpose(t);
end

if size(f,2)==1;
    f=transpose(f);
end

% Mean of times series differences
dt = diff(t);
dt(1:end) = mean(dt);

n = length(f);
y1 = NaN(size(f));

% eq.22b
p = 1;
wt = (3*dt(p) + dt(p+1))/4;
y1(p) = (-f(p+2) + 4*f(p+1) - 3*f(p))/(2*wt);

% eq.22c
p = 2;
wt = (dt(p) + dt(p-1))/2;
y1(p) = (f(p+1) - f(p-1))/(2*wt);

% eq.22a
for p = 3 : n-2
    wt = (8*dt(p) + 8*dt(p-1) + dt(p+1) + dt(p-2))/18;
    y1(p) = (-f(p+2) + 8*f(p+1) - 8*f(p-1) +f(p-2))/(12*wt);
end

% eq.22c
p = n-1;
wt = (dt(p) + dt(p-1))/2;
y1(p) = (f(p+1) - f(p-1))/(2*wt);

% eq.22d
p = n;
wt = (3*dt(p-1) + dt(p-2))/4;
y1(p) = (f(p-2) - 4*f(p-1) + 3*f(p))/(2*wt);
end
```

Published with MATLAB® R2017b
